# Sphingolipids modulate redox signalling during human sperm capacitation

**DOI:** 10.1093/humrep/deae268

**Published:** 2024-12-10

**Authors:** Steven Serafini, Cristian O’Flaherty

**Affiliations:** Experimental Medicine Division, Department of Medicine, McGill University, Montréal, QC, Canada; Urology Division, Department of Surgery, McGill University, Montréal, QC, Canada; The Research Institute, McGill University Health Centre, Montréal, QC, Canada; Experimental Medicine Division, Department of Medicine, McGill University, Montréal, QC, Canada; Urology Division, Department of Surgery, McGill University, Montréal, QC, Canada; The Research Institute, McGill University Health Centre, Montréal, QC, Canada; Department of Anatomy and Cell Biology, McGill University, Montréal, QC, Canada; Department of Pharmacology and Therapeutics, McGill University, Montréal, QC, Canada

**Keywords:** human spermatozoa, nitric oxide, reactive oxygen species, redox signalling, lipid signalling

## Abstract

**STUDY QUESTION:**

What role do sphingolipids have in mediating human sperm capacitation?

**SUMMARY ANSWER:**

Sphingosine 1-phosphate (S1P) mediates the acquisition of fertilizing competency in human spermatozoa by engaging with its Gi-coupled receptor S1PR1 and promoting production of reactive oxygen species such as nitric oxide and superoxide anion.

**WHAT IS KNOWN ALREADY:**

Bioactive sphingolipids, such as S1P, are fundamental for regulating numerous physiological domains and processes, such as cell membranes and signalling, cell death and proliferation, cell migration and invasiveness, inflammation, and central nervous system development.

**STUDY DESIGN, SIZE, DURATION:**

Semen samples were obtained from a cohort of 10 healthy non-smoking volunteers (18–30 years old) to investigate the role of S1P in sperm.

**PARTICIPANTS/MATERIALS, SETTING, METHODS:**

Percoll-selected human spermatozoa were incubated at 37°C for 3.5 h in BWW media with or without foetal cord serum ultrafiltrate (FCSu), sphingosine (Sph), or ceramide (Cer). Spermatozoa were also incubated with or without pharmacological inhibitors of sphingolipid metabolism. Protein tyrosine phosphorylation was determined by immunoblotting. The acrosome reaction was determined by PSA-FTIC labelling of the acrosome and analysed using fluorescence microscopy. Intracellular nitric oxide (NO^•^) production was determined using a DAF-2DA probe. Immunocytochemistry was performed to localize and assess the functional relationship of key components of lipid signalling in spermatozoa. Sperm viability and motility of the samples were evaluated by the hypo-osmotic swelling (HOS) test and computer-aided sperm analysis (CASA). Statistical differences between groups were determined using ANOVA and Tukey’s test. Normal distribution of the data and variance homogeneity were assessed using Shapiro–Wilk and Levene’s test, respectively. A difference was considered significant when the *P*-value was ≤0.05.

**MAIN RESULTS AND THE ROLE OF CHANCE:**

S1P mediates the acquisition of fertilizing competency in human spermatozoa by engaging with its Gi-coupled receptor S1PR1. We found that S1PR1 redistributes to the post-acrosomal region upon induction of capacitation. S1P signalling promotes the activation of the PI3K-AKT pathway, leading to NO^•^ production during sperm capacitation. L-NAME, an nitric oxide synthase inhibitor, impaired the Sph- and Cer-dependent capacitation. Additionally, Sph and Cer promote superoxide anion ($O_{2}^{\mathrm{•-}}$) production, and the extracellular addition of superoxide dismutase (SOD) prevented Sph- and Cer-dependent capacitation, suggesting that Sph and Cer stimulate $O_{2}^{\mathrm{•-}}$ production during sperm capacitation. Protein kinase type R (PKR), ceramide kinase (CERK), and protein kinase C (PKC) are responsible for translocating and activating sphingosine kinase 1 (SphK1), which is necessary to promote S1P production for sperm capacitation.

**LARGE SCALE DATA:**

N/A.

**LIMITATIONS, REASONS FOR CAUTION:**

The utilization and actions of sphingolipids may differ in spermatozoa of different species.

**WIDER IMPLICATIONS OF THE FINDINGS:**

Sphingolipid metabolites such as Sph, Cer, S1P, and ceramide 1-phosphate (C1P) play a crucial role in inducing human sperm capacitation. Our research has provided new insights into fundamental sphingolipid processes in human sperm, including the importance of C1P in translocating and activating SphK1 as well as the S1P signalling to regulate the PI3K/AKT/NOS pathway to generate NO^•^ for sperm capacitation. We are the first to identify the presence of PKR in human spermatozoa and its role in the phosphorylation activities of SphK1 with the subsequent activation of S1P signalling. Furthermore, our study has identified that S1PR1 and S1PR3 are involved in capacitation and the acrosome reaction, respectively. These findings shed light on a novel mechanism by which sphingolipids drive capacitation in human sperm and pave the way for further exploration of the role of bioactive sphingolipid metabolites in this process. Lastly, our studies lay the foundation for examining the lipid profile of infertile males, as potential discrepancies can affect the functional capacity of spermatozoa to reach fertilizing potential.

**STUDY FUNDING/COMPETING INTEREST(S):**

This research was funded by the Canadian Institutes of Health Research (CIHR), grant number PJT-165962 to C.O.F. S.S. was awarded a Research Institute-MUHC Desjardins Studentship. There are no competing interests to report.

## Introduction

Infertility is a prominent health and social concern affecting approximately one in six couples worldwide (Collins, 2019; Cox *et al.*, 2022). Of these infertility cases, over half can be attributed to a male-related factor (Jung and Seo, 2014; Kumar and Singh, 2015). In conjunction, there has been a decline in worldwide semen quality for the past two decades (Aitken, 2013). Despite our continued understanding of the several known causes of male infertility, approximately 34% of cases are classified as idiopathic. These infertile patients have normal sperm analysis results and no other detectable explanation for their inability to achieve clinical pregnancy. The stagnant forward progress in unravelling the causes of idiopathic male factor infertility partly explains the insufficient tools available to accurately assess semen quality and fertilizing capacity. The World Health Organization has established standardized criteria for classifying men based on semen analysis. The criteria focus on sperm concentration, motility, morphology, and ejaculate volume to characterize samples as normozoospermic (World Health Organization, 2021). However, the current semen analysis does not consider the functional capacity of spermatozoa, such as their ability to undergo capacitation. Failure of mature spermatozoa to obtain fertilizing competency could be a cause of idiopathic infertility. Hence, there is room to improve the diagnostic tools for male infertility and to design treatments accordingly.

Ejaculated spermatozoa must reside in the Fallopian tubes to acquire fertilizing ability through the capacitation process and to recognize and fertilize the oocyte (Yanagimachi, 1994). Sperm capacitation is a timely and complex process that encompasses biochemical alterations, including the finely regulated production of reactive oxygen species (ROS), such as superoxide anion ($O_{2}^{\mathrm{•-}}$), hydrogen peroxide and nitric oxide (NO^•^) (de Lamirande and O’Flaherty, 2012; O’Flaherty, 2015), timely regulated phosphorylation events (Serafini and O’Flaherty, 2022), and changes in the plasma membrane lipid contents (Cross, 1998). Capacitation is required to acquire the subsequent hyperactive motility, to recognize and bind the zona pellucida, the calcium-mediated exocytotic release of hydrolytic enzymes known as the acrosome reaction (AR), and ultimately the fertilization of the oocyte (Yanagimachi, 1994; Serafini and O’Flaherty, 2022). The process of sperm capacitation has been known since the 1950s (Austin, 1952), and some proteins (e.g. ion channels, protein kinases, etc.) involved in its molecular mechanism have been described (De Jonge, 2017). However, its regulation, particularly the role of lipid signalling in triggering and regulating capacitation, remains unexplored.

Previously, sphingolipids were thought to have the sole purpose of being a structural component of cell membranes. In recent years, sphingolipids have been shown to play an essential role in membrane biology. They are bioactive signalling lipids that regulate cell motility, growth, senescence, differentiation, and fate (Spiegel and Merrill, 1996; Hannun and Obeid, 2008; Boslem *et al.*, 2012; Newton *et al.*, 2015). These lipids have varying complexity and play a crucial role in regulating physiological pathways. Ceramide (Cer) is synthesized *de novo* from the condensation reaction between palmitate and serine or by hydrolysis of sphingomyelin (SM) by sphingomyelinase (SMase). Ceramide is the hub of sphingolipid metabolism and is converted to sphingosine (Sph) by the action of ceramidase (CDase) or to ceramide 1-phosphate (C1P) through the action of ceramide kinase (CERK). Sphingosine is converted to sphingosine 1-phosphate (S1P) by sphingosine kinase 1 and 2 (SphK1 and SphK2). These bioactive sphingolipid metabolites (Cer, Sph, C1P, and S1P) can act on various targets and regulate multiple physiological pathways. For instance, S1P can regulate physiological pathways by engaging with the S1P-receptor (S1PR) family (Won and Singh, 2006; Vaquer *et al.*, 2020).

The biosynthesis and catabolism of these lipids play an integral role in cellular and physiological functions, including participation in membrane domains and signalling, cell proliferation, death, migration and invasiveness, inflammation, and central nervous system development (Pahan *et al.*, 1998; Singh *et al.*, 1998; Pettus *et al.*, 2002; Proia, 2003; Won *et al.*, 2004; Watterson *et al.*, 2005). Of the total membrane lipids characterized in spermatozoa, sphingolipids constitute about 10–20% (Vaquer *et al.*, 2020). The major sphingolipids in the seminal plasma and spermatozoa are ceramide (Cer) and sphingomyelin (SM) (Vaquer *et al.*, 2020; Furse *et al.*, 2022). Previous reports have shown that S1P in the testis prevents apoptosis of the germ cell line (Suomalainen *et al.*, 2003). In capacitated spermatozoa, Cer (Vaquer *et al.*, 2020), S1P (Suhaiman *et al.*, 2010), and C1P (Vaquer *et al.*, 2023) trigger the acrosome reaction, a controlled exocytosis event necessary for the capacitated spermatozoon to penetrate the zona pellucida and fertilize the oocyte. However, the role of sphingolipids in mediating NO^•^ production during sperm capacitation is unknown.

This study investigated the involvement of sphingolipids in human sperm capacitation. We aimed to identify the molecular signalling pathways activated during this process by following the addition of cell-permeable ceramide and sphingosine.

## Materials and methods

### Materials

Mouse monoclonal anti-tubulin and rabbit polyclonal anti-phospho-PI3K (P-PI3K) antibodies were purchased from Cell Signaling (Beverly, MA, USA). Rabbit polyclonal anti-P-SphK1 antibody was purchased from Invitrogen (Fisher Scientific, Ottawa, ON, Canada). Mouse monoclonal anti-P-Tyrosine (P-Tyr), mouse monoclonal anti-S1PR1 and mouse monoclonal anti-MRP1 antibodies, nitrocellulose blotting membrane, extracellular signal-regulated kinase (ERK) inhibitor (U0126) and protein kinase A (PKA) inhibitor (H89), phospholipase C (PLC) inhibitor (U73122), U73343 (inactive analog of U73122), 1-Oleoyl-2-acetyl-sn-glycerol (OAG), PKR inhibitor, Nω-Nitro-L-arginine methyl ester hydrochloride (L-NAME), progesterone, *Pisum sativum* lectin conjugated with FITC (PSA-FITC), Percoll and Superoxide Dismutase (SOD) from bovine liver were all purchased from Millipore Sigma Canada (Oakville, ON, Canada). SphK1 inhibitor (PF543), CERK inhibitor (NVP231), SPHK2 inhibitor (SLM 6031434), MRP1 inhibitor (MK571), S1PR1/3 inhibitor (VCP 23019), S1PR3 inhibitor (TY 52156), and ceramidase inhibitor (Ceranib-1) were purchased from Tocris Bioscience (Bristol, UK). Horseradish peroxidase-conjugated goat anti-mouse IgG and donkey anti-rabbit IgG antibodies were purchased from Jackson Laboratories (Bar Harbor, ME, USA). Rabbit monoclonal anti-P-PKR and mouse monoclonal anti-CD59 antibodies were purchased from Abcam (Waltham, MA, USA). Pierce ECL Western Blotting Substrate, goat anti-mouse (H+L) and donkey anti-goat (H+L) antibodies, both conjugated with AlexaFluor 555 and AlexaFluor 488, were purchased from ThermoFisher Scientific (Markham, ON, Canada). The enhanced chemiluminescence (ECL) Kit was purchased from ThermoFisher Scientific (Markham, ON, Canada). C6 ceramide (d18:1/6:0), Sphingosine (d18:1), sphingosine-1-phosphate (S1P), and ceramide-1-phosphate (C1P) were purchased from Avanti Polar Lipids Inc. (Alabaster, AL, USA). Protein kinase C (PKC) inhibitor (Chelerythrine) and DAF-2 diacetate (DAF-2DA) were purchased from Cayman Chemicals (Ann Arbor, MI, USA). 2-methyl-6-(p-methoxyphenyl)-3, 7-dihydroimidazo [1, 2-a] pyrazin-3-one (MCLA) and all other chemicals used were of at least reagent grade and purchased from Sigma-Aldrich (Milwaukee, WI, USA). Foetal cord serum samples were obtained from the Cellular Therapy Laboratory, The Research Institute, McGill University Health Centre. Foetal cord ultrafiltrates (FCSu) were prepared with Amicon Ultra-4 filter devices with membranes with an exclusion limit of 3 kDa (MilliporeSigma, Oakville, ON, Canada) as done previously (de Lamirande and Lamothe, 2009).

### Subjects and sperm sample preparation

This study gained approval from the Ethics Board of the Research Institute at the McGill University Health Centre, and all participants gave their informed consent. Semen samples were obtained from healthy donors (18–30 years old) after 72 h of sexual abstinence. Semen samples from different donors were used in each experiment. Samples were incubated for 30 min at 37°C to allow liquefaction. Then, the semen samples were placed on a four-layer Percoll gradient (20%—40%—65%—95%) to separate highly motile spermatozoa found in the 95% Percoll layer and the 65–95% Percoll interface. Sperm motility was analysed using the computer-assisted sperm analysis system from Hamilton Thorne with the software HTCASAII (Beverly, MA, USA). Samples with progressive motility greater than 70% were used. Sperm concentration was assessed using a Neubauer hemacytometer and adjusted to a final concentration of 50 × 10^6^ spermatozoa/ml using Biggers, Whitten, and Whittingham medium (BWW; 91.5 mM NaCl, 4.6 mM KCl, 1.7 mM CaCl_2_, 1.2 mM KH_2_PO_4_, 1.2 mM MgSO_4_, 5.6 mM D-glucose, 0.25 mM sodium pyruvate, 21.6 mM sodium lactate, and 20 mM HEPES, pH 7.95) (Biggers *et al.*, 1971) and incubated with or without 10% foetal cord serum ultrafiltrate (FCSu), a known *in vitro* inducer of human sperm capacitation (de Lamirande and Gagnon, 1993; de Lamirande *et al.*, 1997; Leclerc *et al.*, 1997), or different concentrations of Sph or Cer at 37°C for 3.5 h. FCSu promotes similar changes to those observed when using BSA/bicarbonate (increase of phosphorylation events, hyperactivation, ability of spermatozoa to respond to AR inducers). Moreover, BSA interferes with ROS determination (de Lamirande and Lamothe, 2009) and can impose complications for our ROS quantification experiments. The Sph, Cer, S1P, and C1P working solutions were prepared in bovine serum albumin (BSA) solution in BWW and DMSO. The final concentration of BSA in the samples was 0.12 mg/ml. Samples were also incubated with or without the above-mentioned pharmacological inhibitors. Sperm capacitation was evaluated by the levels of phosphor-tyrosine (P-Tyr) in spermatozoa, P-Tyr is a hallmark of human sperm capacitation (Leclerc *et al.*, 1996).

### Sperm acrosome reaction determination

After incubation for 3.5 h with or without FCSu, Sph or Cer at 37°C, samples were centrifuged at 600×*g* for 5 min, resuspended in regular BWW containing 10 mM progesterone, as described previously (Baldi *et al.*, 1991), and incubated for an additional 30 min at 37°C. Samples were centrifuged at 600×*g* for 5 min, the supernatant was discarded, and the pellet was resuspended in 95% ethanol. Ethanol-fixed sperm (20 µl) was loaded onto Superfrost slides, and without drying, 20 µl of PSA-FITC (30 µg/ml) was added, then incubated at 37°C for 5 min. Slides were then washed with distilled water and dried. A drop of prolonged antifade-DAPI was added, and the slides were covered with a coverslip to determine the percentage of spermatozoa with intact (fluorescence in the acrosome) and reacted acrosomes (lack of fluorescence in acrosome) of 200 spermatozoa per sample using a Zeiss LSM780 Laser Scanning Confocal Microscope (Opti-Tech Scientific, Montreal, QC, Canada) at 100× magnification.

### SDS-PAGE and immunoblotting

- After completion of the 3.5-h incubation, sperm proteins were supplemented with sample buffer containing 100 mM dithiothreitol and phosphatase inhibitors cocktail and boiled for 5 minutes at 100°C. Then, samples were centrifuged at 21 000×*g* for 5 min at room temperature. Samples were loaded onto a 10% acrylamide gel, and electrophoresis was performed for up to 1 h at a constant current of 0.025 A per gel. Following electrophoresis, sperm proteins were electrotransferred into nitrocellulose membranes for 45 min at 100 V. Then, the membranes were blocked in 5% skim milk in tris-buffered saline containing Tween 20 (TTBS) for 1 h. Incubation with primary antibodies was conducted either at room temperature for 1 h (for the anti-P-Tyr antibody (1:10 000v/v)) or overnight at 4°C (for the anti-P-PI3K (1:1000v/v) or anti-P-SphK1 (1:1000v/v) antibodies). After the membranes were washed with TTBS, they were incubated with their respective horse-radish peroxidase-conjugated goat anti-mouse or donkey anti-rabbit antibodies for 45 min at room temperature. The positive immunoreactive protein bands were detected using chemiluminescence. The relative intensities of the protein bands (e.g. for P-Tyr the 105 and 85 kDa bands) for each sample were determined using FIJI Image J (National Institutes of Health, Stapleton, NY, USA) and normalized to that of the loading control obtained with α-tubulin (55 kDa) or silver stain (105 and 85 kDa bands). Protein bands of each sample were secondarily normalized by dividing by the non-capacitated control sample. The protein band relative intensity was expressed as mean ± standard error.

### Immunocytochemistry

We determined the localization and intensity levels of S1PR1, S1PR3, SphK1, and PKR in spermatozoa (permeabilized cells) and the sperm plasma membrane (non-permeabilized and permeabilized cells) as described previously (Liao and O’Flaherty, 2023). Sperm samples were smeared onto Superfrost slides and then dried at room temperature. Cell permeabilization was done by treating sperm samples with 100% methanol for 10 min at −20°C. Nonpermeabilized and permeabilized samples were rehydrated with PBS or PBS + Triton 0.1% (PBS-T) for 20 min. The samples were then incubated in 5% goat serum in fresh PBS or PBS-T for 30 min at room temperature. Primary antibodies were prepared in 5% goat serum PBS and PBS-T and incubated overnight at 4°C. Samples were then washed and incubated with 1:1000 goat anti-mouse secondary antibodies diluted in PBS-T + 1% BSA for 1 h at room temperature. Samples were washed, and a Prolong Antifade with DAPI (Fisher Scientific, Ottawa, ON, Canada) was added before coverslip application. The negative control was incubated with a secondary antibody alone. A magnification of 40× and the same exposure times were used for each sample. FIJI ImageJ software was used for background fluorescence subtraction and quantification of the average relative fluorescence intensity (RFI) of 200 spermatozoa per sample.

### Determination of nitric oxide in spermatozoa by flow cytometry

Nitric oxide was quantified in spermatozoa by flow cytometry using the DAF-2-DA probe. Spermatozoa were diluted to 300 × 10^6^ spermatozoa/ml concentration with Percoll 95% and incubated with 100 µM DAF-2DA for 30 min at 37°C to load the fluorescent probe in the cells. Sample tubes were protected from exposure to light. After this initial incubation, the samples were further diluted with BWW to a final concentration of 50 × 10^6^ spermatozoa/ml and incubated with or without 10% FCSu, 10 μM Sph, or 40 μM Cer and in the presence of S1PR1/3 inhibitor (VCP 23019) or ceramide kinase inhibitor (NVP231), and incubated for 3.5 h at 37°C. After treatment, spermatozoa samples were incubated with 0.2 μM Sytox blue as a viability indicator. Then, spermatozoa were washed and resuspended in 1000 μl of HBS and analysed by flow cytometry. All fluorescence signals of labelled spermatozoa were analysed by a Becton Dickinson flow cytometer FACSCanto II (Becton Dickinson, San Jose, CA, USA) equipped with a 488-nm argon laser as a light source. Nonsperm-specific events were gated out, and a minimum of 20 000 spermatozoa were examined for all conditions at a flow rate of <100 cells/s. The percentage of DAF-2DA^+^ cells with Sytox-blue^+^ cells and the mean fluorescence were calculated and analysed using the flow cytometer software FlowJo version 7.2.2 (FlowJo, Ashland, OR, USA).

### Localization of nitric oxide production in capacitated spermatozoa

Nitric oxide production was localized in spermatozoa by fluorescence microscopy. First, highly motile percoll-selected human spermatozoa at 250 × 10^6^ cells per ml were incubated with 10 μM diaminofluorescein-2 diacetate (DAF-2 DA, a sensitive and specific fluorescent probe for intracellular NO^•^) for 30 min at 37°C (de Lamirande and Lamothe, 2009). The treated samples were further diluted to 30 × 10^6^ and incubated for 3.5 h at 37°C with or without FCSu (10%v/v), Sph (10 μM), and Cer (40 μM). After incubation, sperm samples were centrifuged at 1500×*g* for 5 min, the supernatant was discarded, and the pellet was resuspended in HBS 1× (containing 2% paraformaldehyde). Following a second centrifugation at 1500×*g* for 5 min, the supernatant was removed, and the pellet was resuspended in HBS 1× 2% paraformaldehyde. The sample was loaded onto a Superfrost slide and a drop of Prolong Antifade with DAPI was added before a coverslip. A series of 200 pictures at the same exposure time were taken using a magnification of 100× for each sample with a Leica DM500 microscope (Opti-Tech Scientific, Montreal, QC, Canada) to show NO^•^ production localization in spermatozoa. Levels of NO^•^ were also determined by obtaining the average corrected total cell fluorescence (CTCF) using ImageJ software.

### Determination of superoxide anion production by chemiluminescence

Extracellular $O_{2}^{\mathrm{•-}}$ was determined by MCLA-amplified luminescence using a TECAN M200 Pro Multimode plate reader (Männedorf, ZH, Switzerland) at 5 min intervals, in the integration mode (output summed for 10 s), at 37°C, with constant sample mixing, as described previously (de Lamirande and Gagnon, 1995). Percoll-washed spermatozoa at 30 × 10^6^ cells/ml were loaded onto a Nunclon™ Delta Surface plate, with BWW media, and with or without FCSu, Sph, and Cer. Chemiluminescence measurements were taken immediately upon the addition of 20 μM MCLA. MCLA is a very sensitive and cell-impermeant probe. It is important to run each sample alongside a similar sample that has been supplemented with SOD. This allowed us to consider only the SOD-inhibitable (SOD-inh) signal. Additionally, it is important to test appropriate blanks (incubation medium supplemented or not with SOD) in parallel, as the incubation medium itself and the substances being tested may also affect the observed chemiluminescence.

### Statistical analysis

All data were shown as mean ± SEM and statistical differences between groups were determined using ANOVA and Tukey’s test using GraphPad Prism 5 (GraphPad Software, Inc., San Diego, CA, USA). Normal distribution of the data and variance homogeneity were assessed using Shapiro–Wilk and Levene’s test, respectively. A difference was considered significant when the *P*-value was ≤0.05.

## Results

### Sphingosine (Sph) and ceramide (Cer) induce sperm capacitation

**Figure 1.**
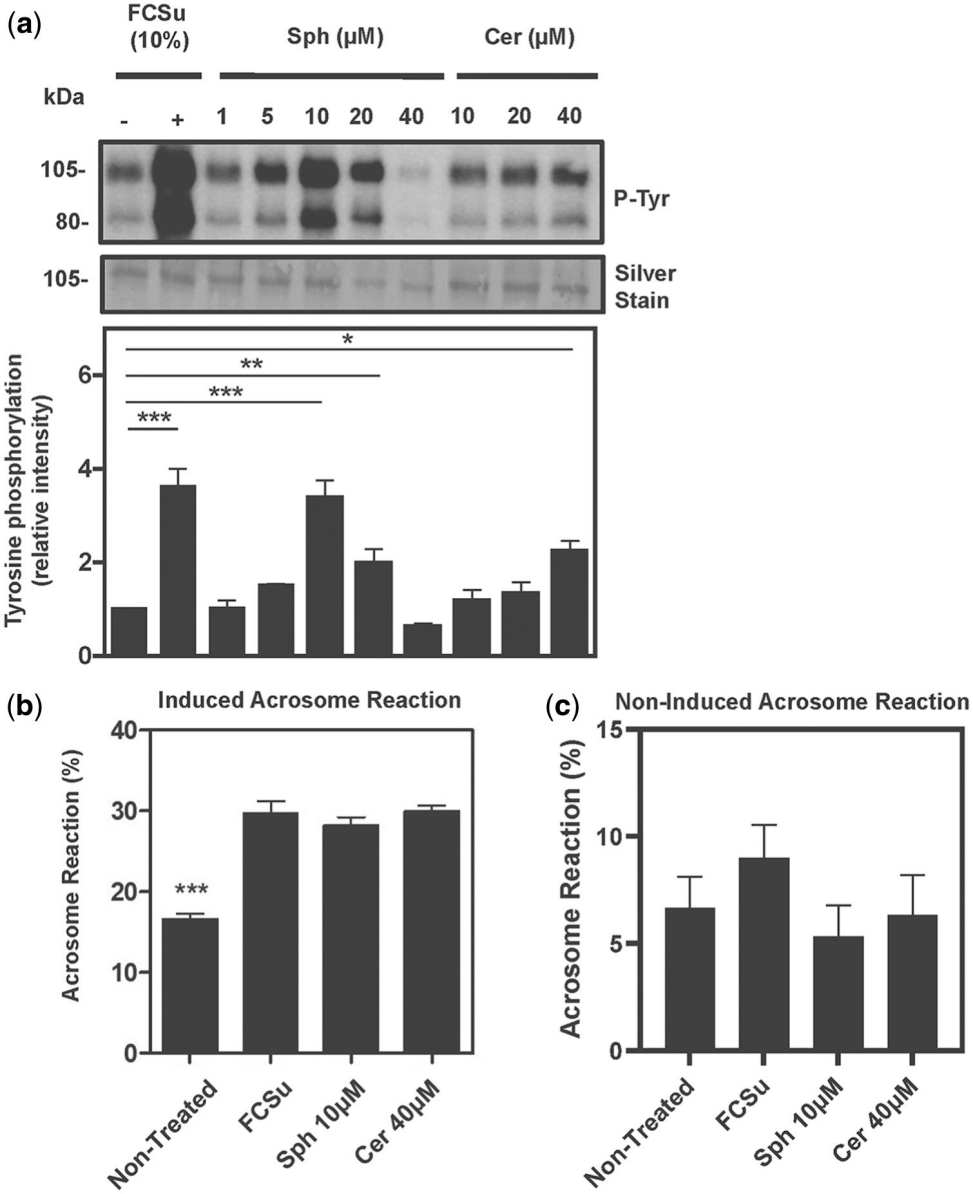
**Sph and Cer induce sperm capacitation.** (**a**) Phospho-tyrosine (P-Tyr) intensity of 80- and 105-kDa proteins in spermatozoa treated with FCSu, sphingosine (Sph), or ceramide (Cer) for 3.5 h at 37°C. (**b**) Progesterone-induced acrosome reaction (measured as a % of spermatozoa without intact acrosome) in spermatozoa pre-treated in BWW medium with or without control inducer FCSu (10%v/v), 10 μM (Sph), and 40 μM Cer. (**c**) Non-induced acrosome reaction in spermatozoa pre-treated in BWW medium with or without control inducer FCSu (10%v/v), Sph (10 μM), and Cer (40 μM). After incubation, spermatozoa samples were or were not treated with 10 μM of progesterone to induce the acrosome reaction. Samples treated with sphingolipids increased the percentage of spermatozoa that underwent the induced acrosome reaction as compared with non-capacitated samples (b). Samples not incubated with progesterone showed no change in acrosome exocytosis percentage compared to the non-treated controls (c). The results represent sperm samples from different healthy donors (n = 4, ANOVA and Tukey test; *P≤0.05; **P≤0.01; ***P ≤ 0.001). For all experiments on acrosome reaction, 200 cells were evaluated for each sample.

We found that both Sph and Cer increased P-Tyr levels (Fig. 1a), and the progesterone-induced acrosome reaction (Fig. 1b) compared to those observed in non-treated controls. The levels of progesterone-induced acrosome reaction observed in spermatozoa treated with both sphingolipids were comparable to those obtained when spermatozoa were treated with FCSu. In addition, Sph and Cer did not result in the spermatozoa undergoing spontaneous acrosome reaction (Fig. 1c). These results indicated that sphingosine and ceramide promote human sperm capacitation *in vitro*. Since the highest levels of P-Tyr were obtained with 10 µM Sph and 40 µM Cer, we used these concentrations for further experimentation.

### Conversion of ceramide to sphingosine by ceramidase (CDase) is fundamental for capacitation

We took one step further and assessed whether the conversion of Cer to the dominant metabolite Sph is fundamental for capacitation. We found that FCSu- and Cer-treated spermatozoa incubated with Ceranib-1 (CDase inhibitor) at 40 or 50 μM had lower P-Tyr levels than those cells incubated without the inhibitor (Supplementary Fig. S1). This indicates the importance of the sequential generation of sphingolipid metabolites and that the post-translational modification of P-Tyr during capacitation cannot proceed without the generation of Sph.

### Involvement of sphingosine kinase 1 (SphK1) and ceramide kinase (CERK) is required for sperm capacitation

**Figure 2.**
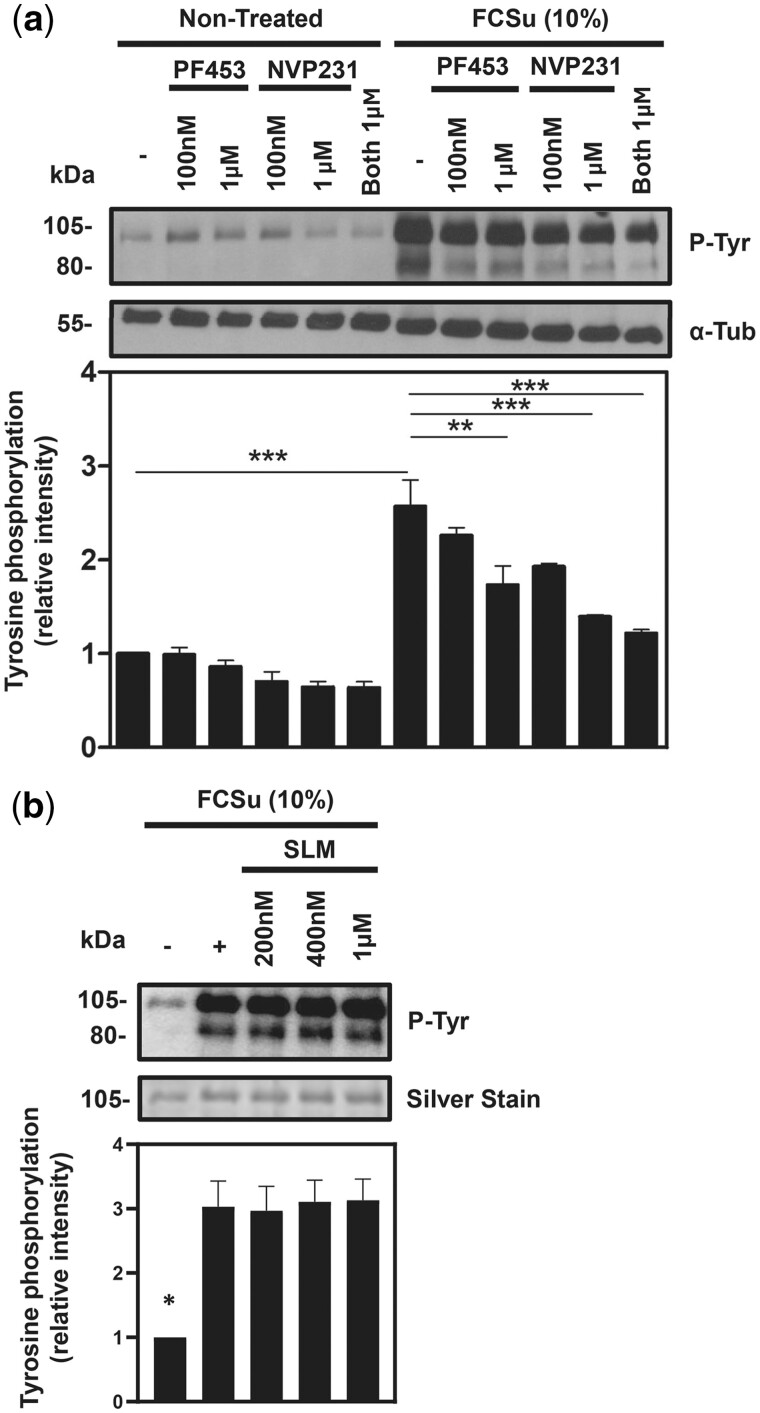
**The production of sphingosine 1-phosphate (S1P) and ceramide 1-phosphate (C1P) is vital for sperm capacitation.** Effect of PF453 (SphK1 inhibitor), NVP213 (CERK inhibitor), and SLM (SphK2 inhibitor) on tyrosine phosphorylation (P-Tyr) (**a** and **b**) and phospho-SphK1 (P-SphK1) fluorescence (**c**). Spermatozoa were incubated for 3.5 h under capacitating conditions without any treatment (control) or the presence of FCSu (10% v/v), with or without PF453, NVP213, and SLM. (a) The immunoblotting analysis shows decreased levels of P-Tyr in human spermatozoa treated with increasing concentrations (100 nM and 1 μM) of SphK1 and CERK inhibitors. (b) Inhibition of SphK2 with SLM did not change the levels of P-Tyr. The results represent sperm samples from different healthy donors (n = 4, ANOVA and Tukey test; *P≤0.05; **P≤0.01; ***P ≤ 0.001).

Having established the importance of Sph and Cer during capacitation, we shifted our attention towards the kinases SphK1/2 and CERK and their respective production of S1P and C1P. We studied the effects of PF543 (a selective inhibitor of SphK1), NVP231 (a specific CERK inhibitor), or SLM 6031434 (an inhibitor that has 40-fold higher selectivity for SphK2 over SphK1) during sperm capacitation. Non-treated and FCSu-treated spermatozoa were incubated with increasing concentrations (100 nM and 1 μM) of PF543 and NVP231, leading to a dose-dependent decrease in P-Tyr levels compared to the untreated controls (Fig. 2a). In contrast, SLM did not impair P-Tyr levels, suggesting that SphK2 is not involved in sphingolipid metabolism during capacitation (Fig. 2b).

**Figure 3.**
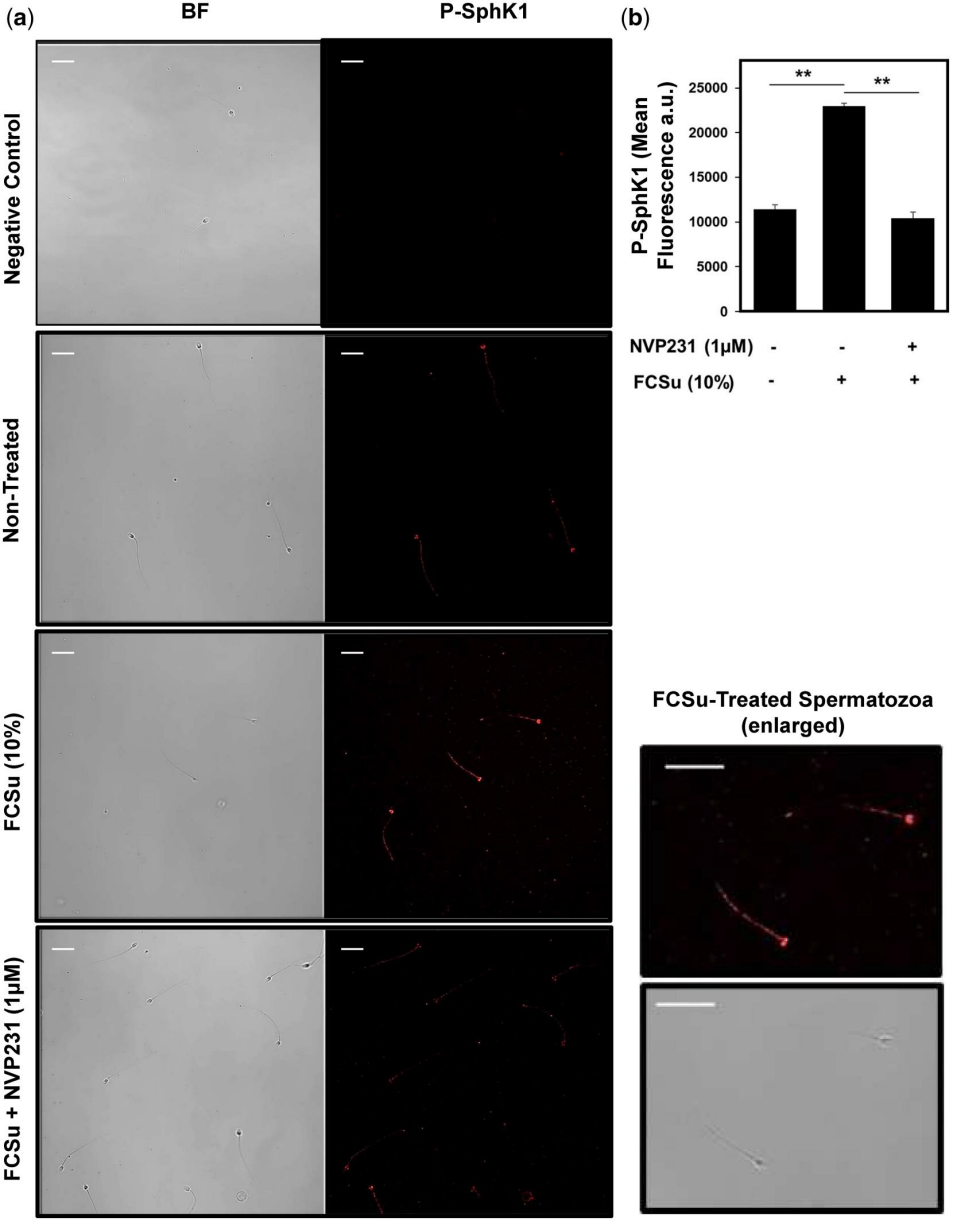
**Ceramide 1-phosphate (C1P) is required for the activation of SphK1 and sphingosine 1-phosphate (S1P) production.** (**a**) Immunocytochemistry analysis of permeabilized spermatozoa showed the increase in fluorescence intensity of P-SphK1 with FCSu and diminished signal following inhibition with NVP231 (scale bar = 20 μM). (**b**) For the mean fluorescence intensity in arbitrary unit (a.u.), 200 cells were evaluated in the permeabilized samples. The results represent sperm samples from different healthy donors (n = 4, ANOVA and Tukey test; **P≤0.01).

SphK1 is predominantly cytosolic and is recruited to the inner leaflet of the plasma membrane (Gault *et al.*, 2010), where it is phosphorylated at the Ser-225 residue and activated. Phosphorylated SphK1 (P-SphK1) can then convert Sph into S1P. Reports have surfaced that numerous co-factors, including C1P, support the recruitment of SphK1 to the plasma membrane (Nishino *et al.*, 2019). To verify whether this occurs in human spermatozoa, we treated spermatozoa with 10% FCSu in the presence or absence of a CERK inhibitor (NVP231) and assessed SphK1 phosphorylation by immunocytochemistry. We first observed that in permeabilized spermatozoa, P-SphK1 localizes to the post-acrosomal region and faintly in the flagellum, and the relative intensity of this phosphorylation increased in FCSu-capacitated compared to non-treated controls (Fig. 3a). Moreover, the inhibition of CERK with NVP231, which impairs C1P production, led to a decrease in P-SphK1 levels in FCSu-treated spermatozoa, similar to the levels observed in non-treated controls (Fig. 3b).

### Sphingosine 1-phosphate with extracellular transport by ABCC1, but not extracellular ceramide 1-phosphate (C1P), induce sperm capacitation

**Figure 4.**
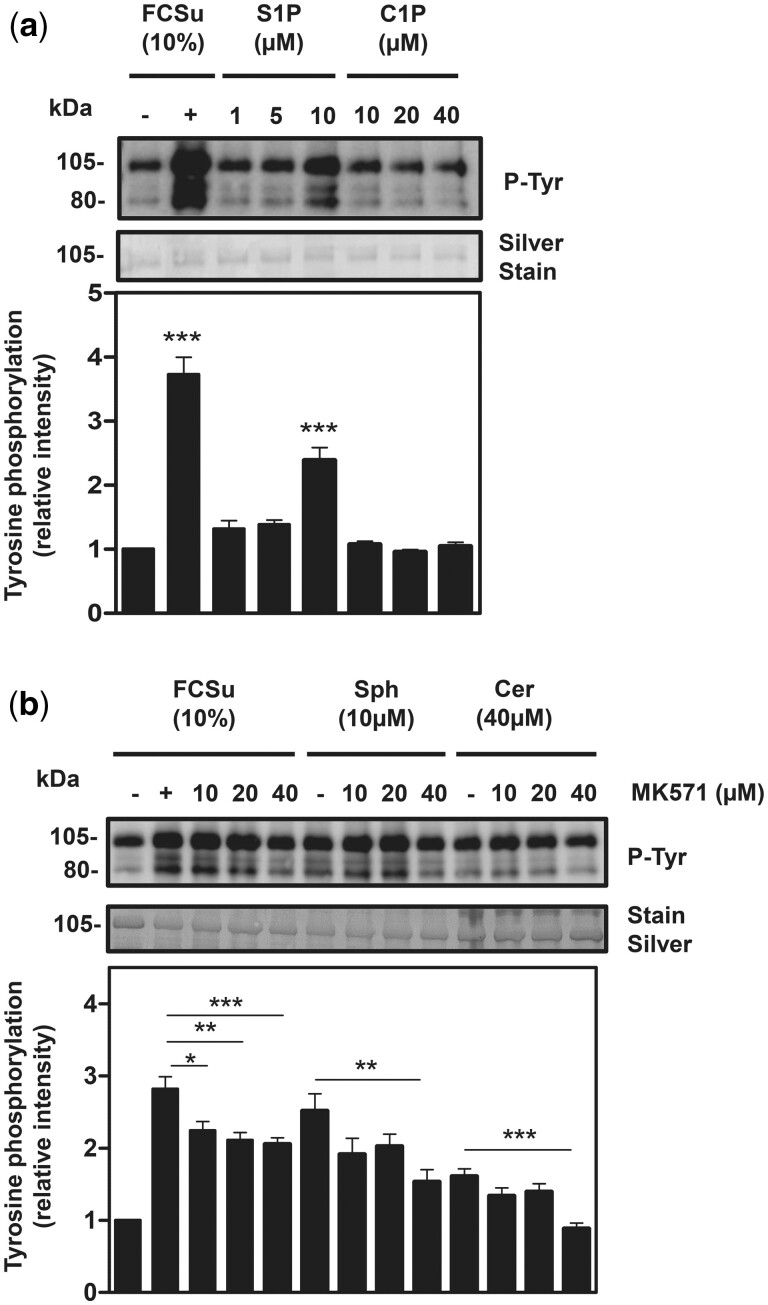
**S1P produced intracellularly requires ABCC1-transport to reach the extracellular leaflet and engage with S1P-receptor family.** Effect of sphingosine 1-phosphate (S1P), ceramide 1-phosphate (C1P), sphingosine (Sph), ceramide (Cer), and MK571 (ABCC1 transporter inhibitor) on tyrosine phosphorylation (P-Tyr). Spermatozoa were incubated for 3.5 h under capacitating conditions without any treatment (control) or the presence of FCSu, S1P, C1P, Sph, or Cer with or without MK571. (**a**) The immunoblotting analysis shows that upon increasing dosage, P-Tyr only increased with 10 μM S1P and not with other concentrations of S1P or C1P (10, 20, 40 μM). (**b**) The immunoblotting analysis demonstrates that a 40-μM concentration of MK571 decreased P-Tyr in samples capacitated with FCSu, Sph, and Cer. The results represent sperm samples from different healthy donors (n = 4, ANOVA and Tukey test; *P≤0.05; **P≤0.01; ***P ≤ 0.001).

The fact that inhibitors of SphK1 and CERK prevented the rise in P-Tyr levels in FCSu-treated spermatozoa indicates that these kinases are necessary for supporting capacitation. The next step was to assess whether extracellular S1P and C1P can promote capacitation-associated modifications. We observed higher levels of P-Tyr when spermatozoa were treated with 10 µM S1P but not with C1P (at all concentrations tested) as compared to non-treated controls (Fig. 4a), validating the participation of S1P signalling during capacitation. Intracellular S1P produced by SphK1 to engage with its receptor requires transport from the inner leaflet to the outer leaflet of the membrane. One of the possible transporters would be an ATP-binding cassette subfamily C member 1 (ABCC1) transporter (Suhaiman *et al.*, 2010). The ABCC1 inhibitor MK571 prevented the FCSu-, Sph-, or Cer-dependent increase of P-Tyr in spermatozoa (Fig. 4b), implying that S1P shuttling to the extracellular leaflet by ABCC1 occurs during capacitation.

### S1PR1 is essential for S1P signalling through PI3K during sperm capacitation, while S1PR3 is essential for mediating the acrosome reaction

**Figure 5.**
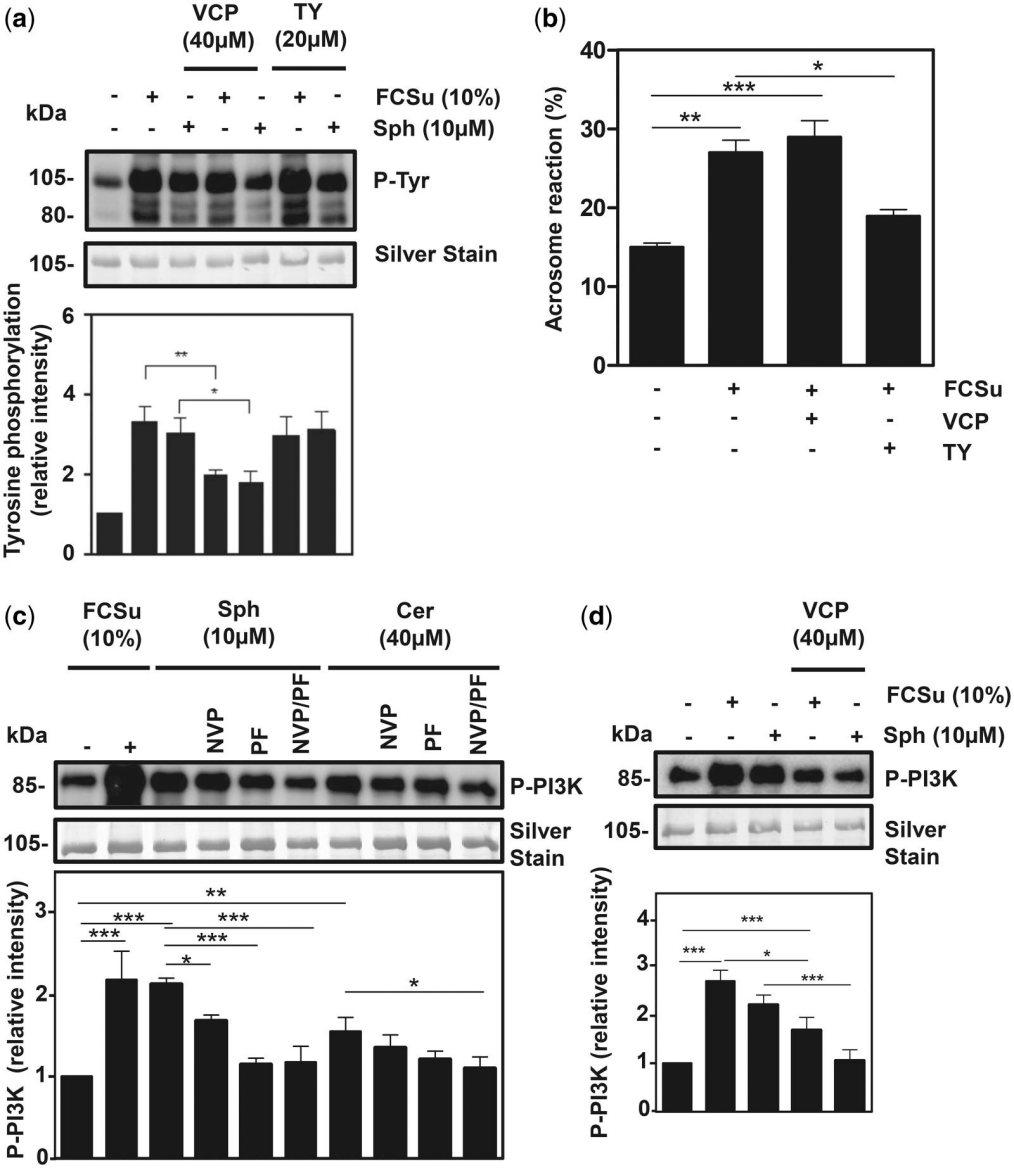
**S1PR1-mediated S1P signalling mediates sperm capacitation by activating the PI3K pathway.** Effect of VCP23019 (S1PR1 inhibitor) and TY52156 (S1PR3 inhibitor) on tyrosine phosphorylation (P-Tyr), PI3K phosphorylation (P-PI3K), and acrosome exocytosis (absence of FITC labelling). Spermatozoa were incubated for 3.5 h under capacitating conditions without any treatment (control) or the presence of foetal cord serum ultrafiltrate (FCSu), sphingosine (Sph), with or without VCP23019 and TY52156. For acrosome reaction, spermatozoa were capacitated with or without FCSu for 3.5 h. After they were supplemented with or without VCP23019 and TY52156 for 15 min before the addition of progesterone. Then progesterone at (10 μM) was added and incubated for 30 min to induce the acrosome reaction. (**a**) The immunoblotting analysis demonstrated that a 40 μM concentration of VCP23019 decreased P-Tyr in samples capacitated with FCSu and Sph, but not TY52156. (**b**) Acrosome exocytosis analysis demonstrated that only the addition of TY52156 reduced the number of cells that underwent acrosome reaction. For all experiments on acrosome reaction, 200 cells were evaluated for each sample. The immunoblotting analysis shows that (**c**) PF543 and NVP231 and (**d**) VCP23019 decreased P-PI3K in both Sph-, ceramide (Cer)-, and FCSu-treated spermatozoa. The results represent sperm samples from different healthy donors (n = 4, ANOVA and Tukey test; *P≤0.05; **P≤0.01; ***P ≤ 0.001).

The sphingosine-1-phosphate (S1P) family of G protein-coupled receptors, comprising five members, S1PR1, S1PR2, S1PR3, S1PR4, and S1PR5, are essential in mediating S1P signal transduction in numerous cellular functions, e.g. proliferation, migration, and cytoskeleton organization (Sanchez and Hla, 2004). In human testis, only two of the five members, S1PR1 and S1PR3, are expressed (Djureinovic *et al.*, 2014). Our goal was to understand which receptor is necessary to transmit S1P signal transduction during sperm capacitation. We observed that the inhibition of S1PR1 by VCP23019, but not that of S1PR3 by TY52156 (TY), decreased P-Tyr levels promoted by FCSu (Fig. 5a). These results indicate that S1PR1, and not S1PR3, is essential for regulating lipid signalling during human sperm capacitation.

To further unravel the role of S1PR3 in spermatozoa, we determined whether its pharmacological inhibition impairs the progesterone-induced acrosome reaction in pre-capacitated spermatozoa. It is known that S1P induces calcium-mediated acrosome exocytosis, yet the receptor involved has not been identified (Suhaiman *et al.*, 2010). Our findings demonstrated that the inhibition of S1PR3, but not of S1PR1, prevented the acrosome reaction in spermatozoa previously capacitated with FCSu or Sph (Fig. 5b), suggesting that both receptors are differentially regulated and serve essential roles during the processes leading up to the fertilization of the oocyte (Suhaiman *et al.*, 2010).

As mentioned previously, the PI3K pathway is a major contributor to the capacitation process in humans (Luconi *et al.*, 2004), yet its regulation has not been fully elucidated. First, we assessed whether S1P and C1P production leads to the activation of the PI3K-AKT pathway. Impairing S1P and C1P production when incubating spermatozoa with or without Sph and Cer and pharmacologically inhibiting SphK1 (1 μM of PF543) and CERK (1 μM of NVP231) led to a decrease of P-PI3K (Fig. 5c). Then, to assess whether S1PR1-mediated S1P signalling facilitates capacitation by activating the PI3K pathway, we incubated spermatozoa with or without capacitation inducers (FCSu and Sph) and VCP23019. VCP23019 led to lower levels of P-PI3K in FCSu- and Sph-treated spermatozoa compared to untreated controls (Fig. 5d), indicating that S1PR1-mediated S1P signalling activates the PI3K pathway.

**Figure 6.**
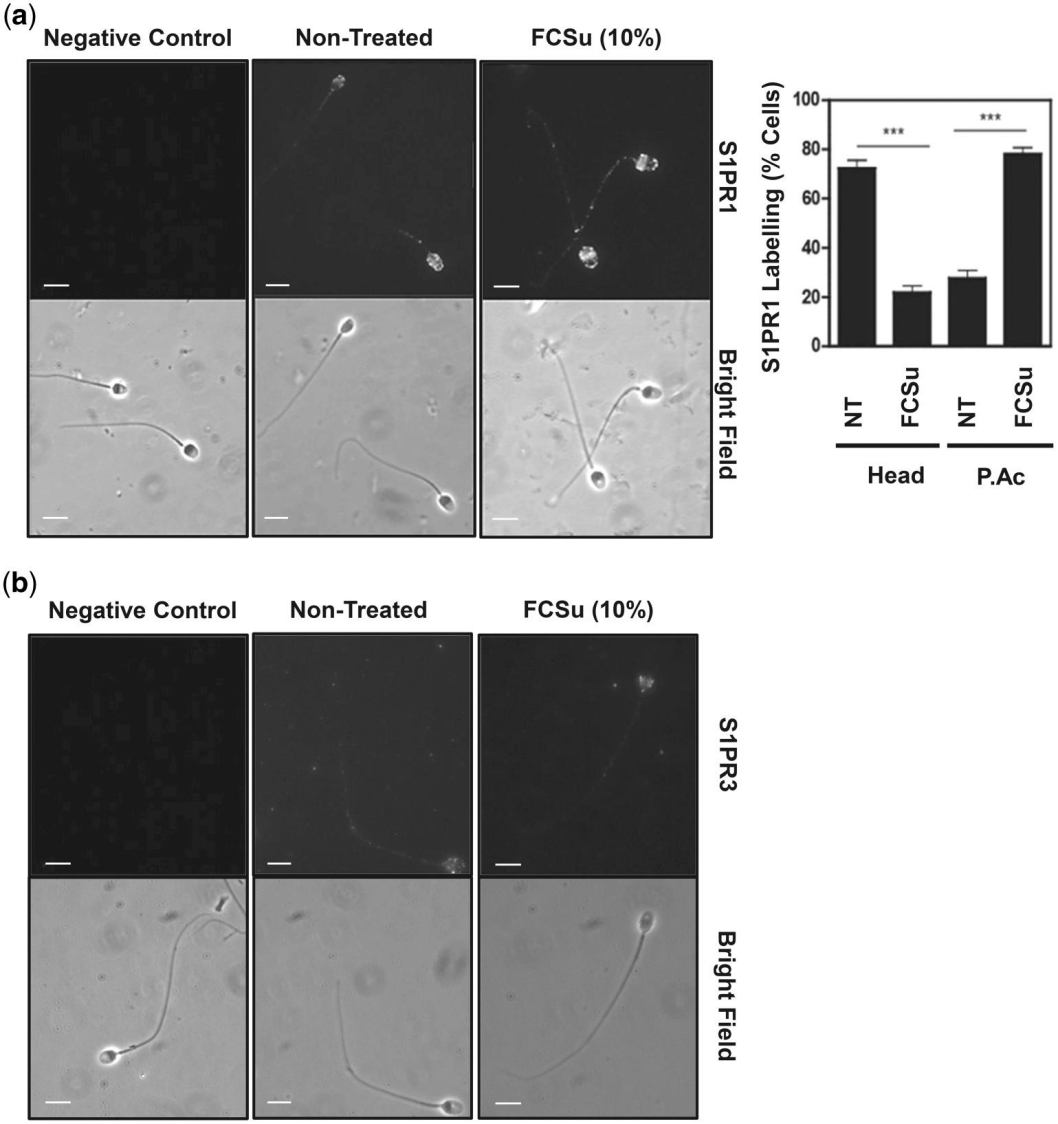
**Localization of S1PR1 and S1PR3 in capacitated spermatozoa.** Spermatozoa were incubated for 3.5 h under capacitating conditions without any treatment (control) or the presence of foetal cord serum ultrafiltrate (FCSu). Samples were added to super-frosted slides and exposed to anti-S1PR1 or anti-S1PR3. (**a**) Immunocytochemistry analysis showed an altered distribution of anti-S1PR1 between control and capacitated conditions (scale bar = 5 μM). (**b**) Immunocytochemistry analysis showed no change in the distribution of anti-S1PR3 between control and capacitated conditions (scale bar = 5 μM). For immunocytochemistry analysis, 200 cells were analysed per permeabilized sample. The results represent sperm samples from different healthy donors (n = 4, ANOVA and Tukey test; ***P ≤ 0.001).

Next, we performed immunocytochemistry using an anti-S1PR1 and anti-S1PR3 antibody in spermatozoa treated with or without FCSu to localize the two receptors in human spermatozoa. We observed that the non-treated permeabilized spermatozoa had widespread distribution of the S1PR1 signal throughout their heads, while FCSu-treated permeabilized spermatozoa underwent a re-localization of the S1PR1 signal towards the post-acrosomal region and equatorial segment of the sperm head (Fig. 6a). Furthermore, S1PR3 was found in the post-acrosome region in both non-treated and FCSu-treated samples (Fig. 6b).

### S1PR1-mediated S1P signalling is associated with ERK, PKC, and PLC but not PKA

We assessed whether the activation of other pathways is associated with S1P signalling. The inhibition of PKC with 10 μM Chelerythrine and extracellular signal-regulated kinase (ERK)1 and 2 with 10 μM U0216 (Supplementary Fig. S2a and b) in FCSu-, Sph-, and Cer-treated spermatozoa led to a decrease in P-Tyr levels. Moreover, inhibition of the protein kinase A (PKA) pathway with 10 μM H89 (O’Flaherty *et al.*, 2005) in the FCSu-treated sample impaired P-Tyr but not in Sph-, and Cer-treated samples (Supplementary Fig. S2c). This suggests that sphingolipid signalling does not engage with the protein kinase A (PKA) pathway during capacitation. Moreover, we investigated the activation of phospholipase C (PLC) by sphingolipid signalling. FCSu-, Sph-, and Cer-treated spermatozoa with or without 2.5 μM of phospholipase C (PLC) inhibitor (U73122) decreased P-Tyr levels (Supplementary Fig. S2d). U73343, an inactive analog of U73122, did not modify the P-Tyr levels in FCSu-treated spermatozoa (Supplementary Fig. S2e). Additionally, overriding the PLC inhibition with 1-Oleoyl-2-acetyl-sn-glycerol (OAG) prevented the reduction in P-Tyr levels (Supplementary Fig. S2d).

### Sphingosine and ceramide promote nitric oxide (NO^•^) and superoxide ($O_{2}^{\mathrm{•-}}$) production in human spermatozoa

**Figure 7.**
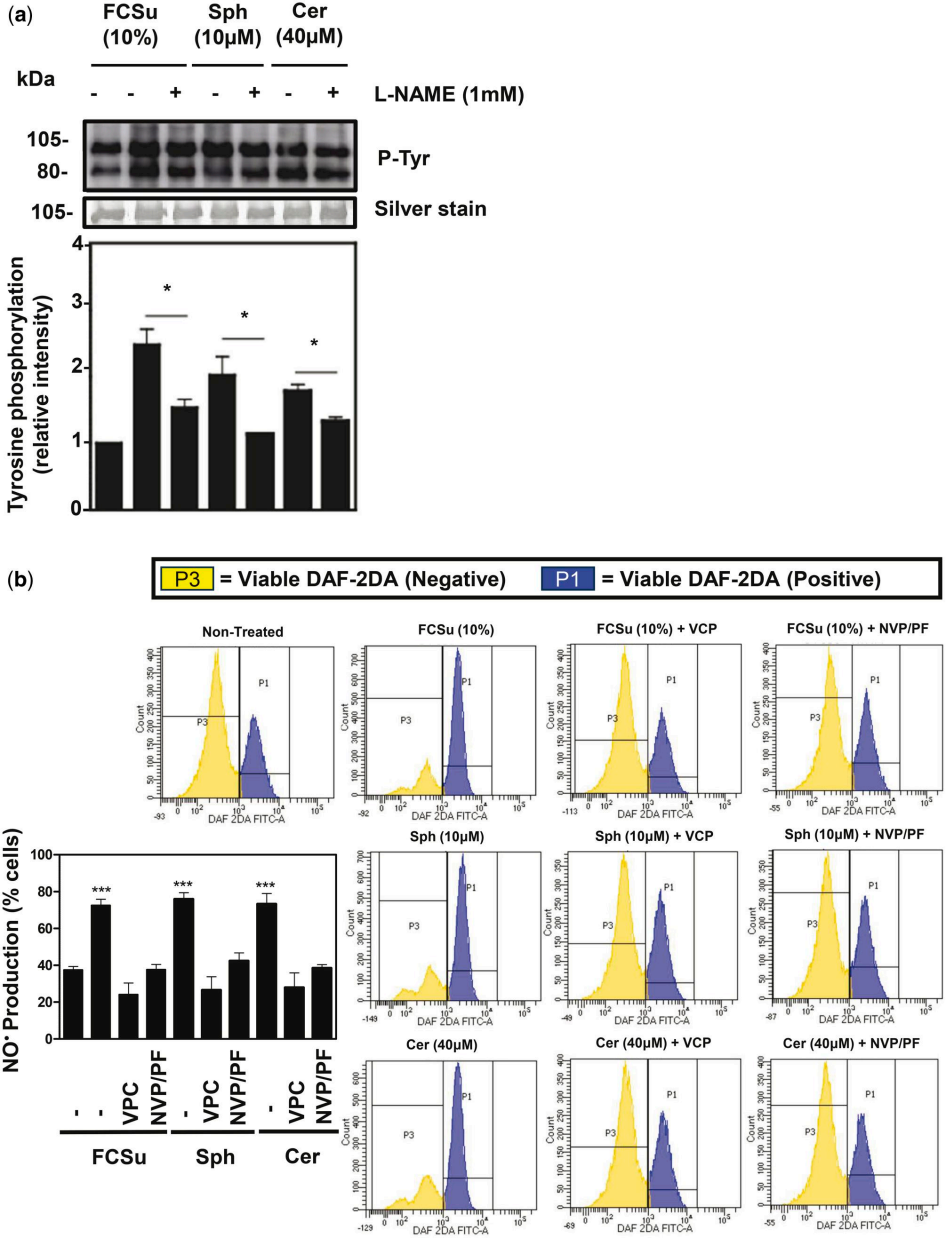
**Sphingolipids stimulate NO^•^ production during capacitation.** Foetal cord serum ultrafiltrate (FCSu)-, sphingosine (Sph)-, and ceramide (Cer)-capacitated spermatozoa incubated with or without L-NAME (nitric oxide synthase ‘NOS’ inhibitor), NVP231 (CERK inhibitor), PF543 (SphK1 inhibitor), and VCP23019 (S1PR1 inhibitor), on tyrosine phosphorylation (P-Tyr) and DAF-2DA (probe for intracellular nitric oxide (NO^•^)) and Sytox Blue (viability dye) fluorescence. (**a**) The immunoblotting analysis shows a decrease in P-Tyr with L-NAME. Each lane was normalized to its silver-stain optical density value for signal quantification. (**b**) Histogram of viable spermatozoa (Sytox Blue negative) that are either DAF2-DA^+^ (blue, P1) or DAF2-DA^−^ (yellow, P3) for NO^•^ production. These spermatozoa were incubated with the inducers FCSu, Sph, and Cer, and were treated with VCP23019, NVP231, and PF543. The results represent sperm samples from different healthy donors (n = 4, ANOVA and Tukey test; *P≤0.05; ***P ≤ 0.001).

Phosphorylation of PI3K leads to the activation of AKT, a kinase that phosphorylates a variety of substrates, including the nitric oxide synthase (NOS), an enzyme present in spermatozoa (Herrero *et al.*, 1996; O’Bryan *et al.*, 1998) and involved in human sperm capacitation (Nauc *et al.*, 2004; O’Flaherty *et al.*, 2006a,b). However, the regulation of NOS in spermatozoa remains unknown. We opted to further unravel its regulation by capacitating spermatozoa with or without FCSu or Sph in the presence or absence of 1 mM of L-NAME, a cell-permeable inhibitor of NOS that prevents human sperm capacitation (de Lamirande and Gagnon, 1995; Aitken *et al.*, 1997; de Lamirande *et al.*, 1997; Herrero *et al.*, 1999; Olds-Clarke, 2003; Thundathil *et al.*, 2003; De Jonge, 2005; de Lamirande and Lamothe, 2009). We observed decreased P-Tyr levels in FCSu- or Sph-treated spermatozoa compared to their controls without L-NAME (Fig. 7a). We also localized NO^•^ production using fluorescence microscopy by loading spermatozoa with DAF2-DA. This probe emits fluorescence in the presence of intracellular production of this free radical. The NO^•^ production by FCSu, Sph, and Cer was found in the post-acrosomal region of the spermatozoa head, but not in the non-treated controls (Supplementary Fig. S3).

Next, using flow cytometry, we determined the number of viable spermatozoa that produced NO^•^ (Sytox blue) and assessed whether this production requires the participation of S1PR1, SphK1, and CERK. We incubated spermatozoa with FCSu, Sph, or Cer and with or without VCP23019, PF543 and NVP 231. We observed an increase in NO^•^ in FCSu-, Sph-, and Cer-treated samples compared to the non-treated condition, as depicted by the greater number of cells in the DAF-2DA^+^ (blue, P1) compared to the DAF-2DA^−^ (yellow, P3) (Fig. 7b). NO^•^ production by the inducers was diminished (increase in yellow, P3) by the inhibition of S1PR1 and SphK1/CERK (Fig. 7b and Supplementary Fig. S4). This result indicates that Sph and Cer signalling yields intracellular NO^•^, a major player in human sperm capacitation (Herrero, de Lamirande and Gagnon, 1999; de Lamirande and O’Flaherty, 2012).

**Figure 8.**
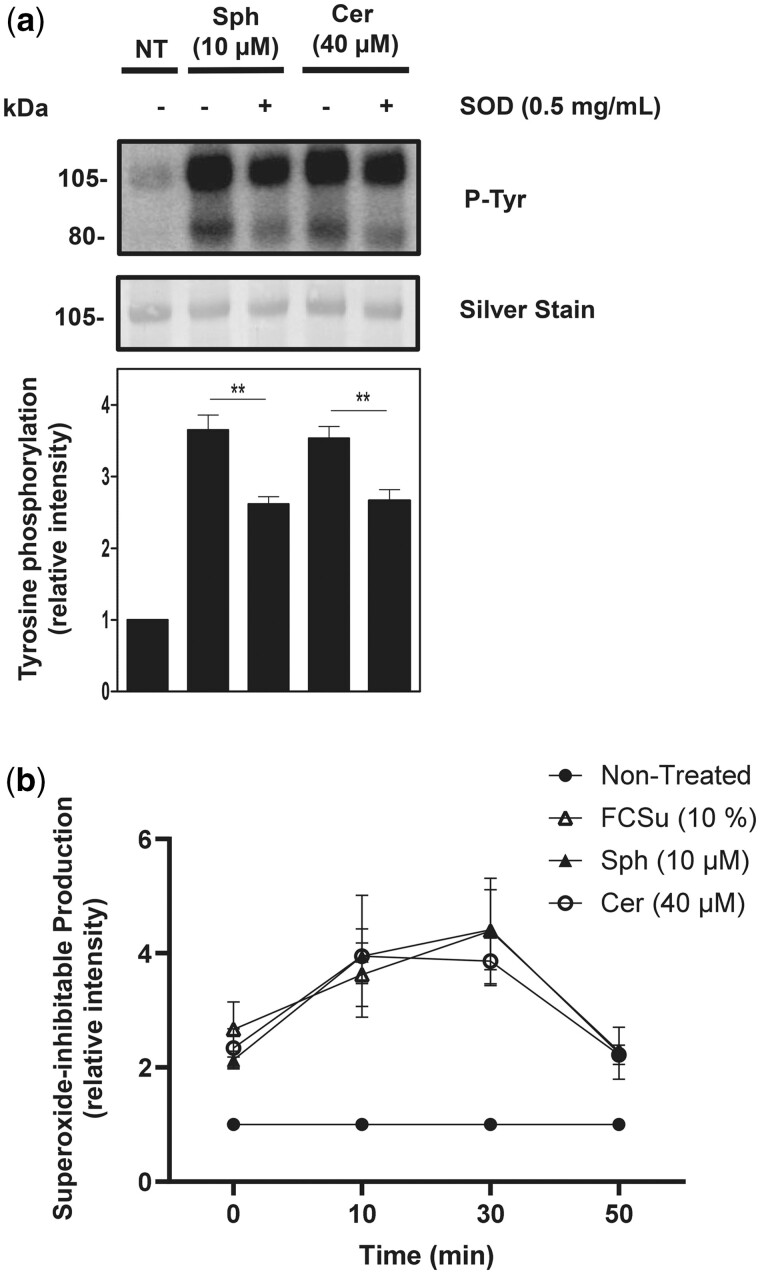
**Sphingolipids stimulate superoxide production during capacitation.** Foetal cord serum ultrafiltrate (FCSu)-, sphingosine (Sph)-, and ceramide (Cer)-capacitated spermatozoa incubated with or without superoxide dismutase (SOD). (**a**) P-Tyr immunoblot of samples incubated with Sph and Cer and 0.5 mg/ml of SOD. (**b**) Spermatozoa were incubated in BWW supplemented with or without FCSu (10%, v/v) (-Δ-), Sph (10 μM) (-Δ-), and Cer (40 μM) (-○-) in the absence or presence of SOD (0.5 mg/ml), and chemiluminescence was measured after the addition of MCLA (20 μM). The data shown represent the relative intensity (standardized to non-treated) signal of SOD-inh chemiluminescence. The results represent sperm samples from different healthy donors (n = 4, ANOVA and Tukey test; **P ≤0.01).

To further elucidate the involvement of sphingolipids in redox signalling, we assessed whether Sph and Cer promote $O_{2}^{\mathrm{•-}}$ production. Spermatozoa treated with Sph or Cer in the presence of 0.5 mg/ml SOD displayed lower levels of P-Tyr compared to those treated in the absence of SOD (Fig. 8a). Moreover, quantification of $O_{2}^{\mathrm{•-}}$ production showed a 4-fold increase in FCSu-, Sph-, and Cer-treated samples compared to non-treated conditions (Fig. 8b). Hence, sphingolipids are involved in the $O_{2}^{\mathrm{•-}}$ production during sperm capacitation.

### Activation of SphK1 by PKC and PKR during sperm capacitation

Following the translocation of SphK1 to the inner leaflet of the plasma membrane, this kinase is activated by phosphorylation at Ser225 (Pitson *et al.*, 2003). Thus, we evaluated what kinase is responsible for SphK1 phosphorylation during human sperm capacitation. One of the candidates is PKC, which is involved in human sperm capacitation (Furuya *et al.*, 1993; O’Flaherty *et al.*, 2005). We first demonstrated that FCSu, Sph, and Cer caused the activation of PLC to activate PKC (Supplementary Fig. S2a and d). Then, we assessed whether the activated PKC can subsequently activate SphK1. The treatment of FCSu-incubated spermatozoa with U73122 decreased the P-Tyr and P-SphK1 levels, and the addition of OAG prevented the reduction of their respective intensities (Supplementary Figs S2d and S5a). Then, we incubated spermatozoa with FCSu and Chelerythrine and observed a decrease in both P-SphK1 and P-Tyr levels (Supplementary Fig. S5b). Thus, the PLC/PKC pathway mediates the S1P signalling during sperm capacitation.

**Figure 9.**
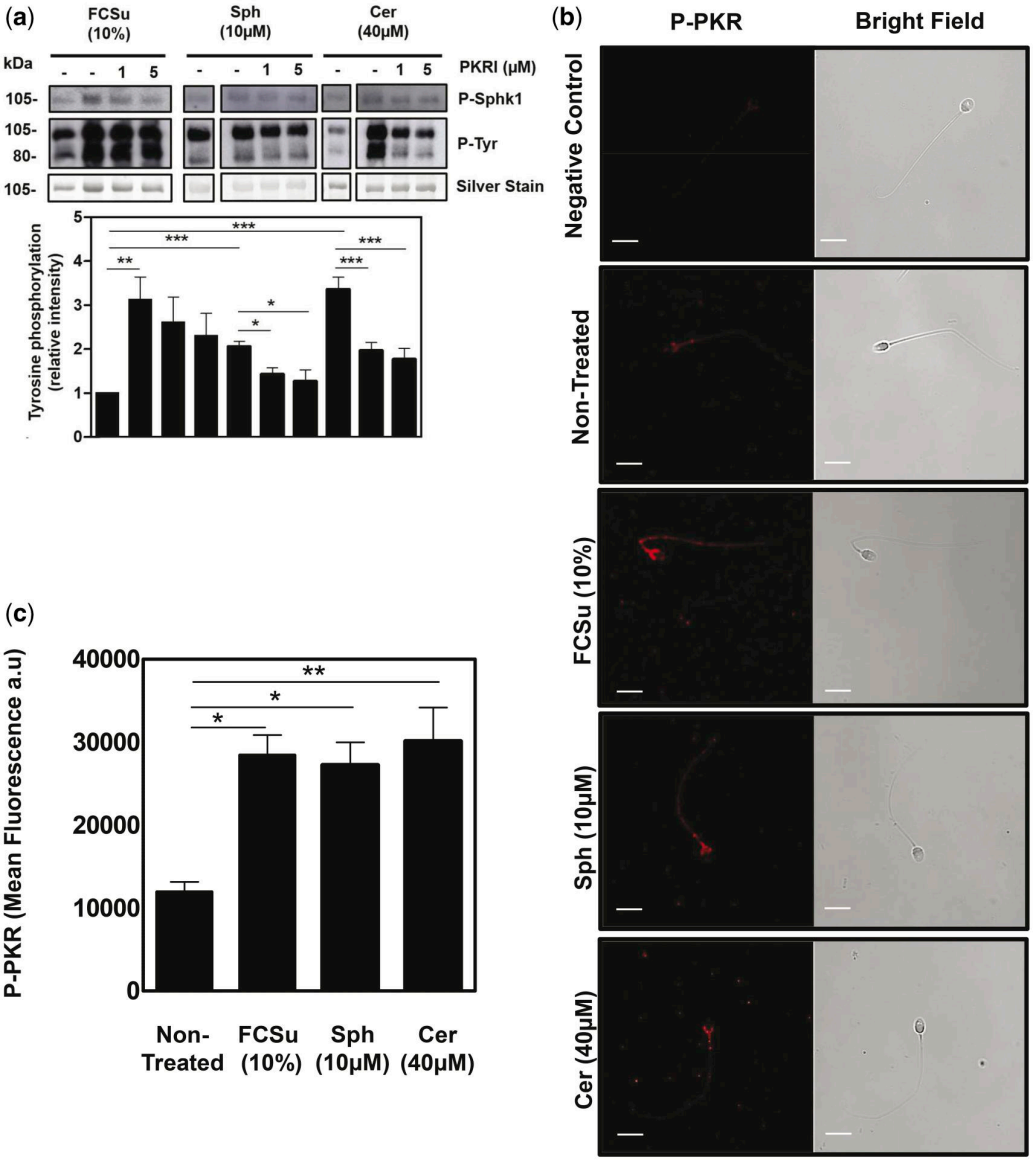
**PKR activates SphK1 during human sperm capacitation.** Foetal cord serum ultrafiltrate (FCSu)-, sphingosine (Sph)-, and ceramide (Cer)-capacitated spermatozoa incubated with or without protein kinase R (PKR) inhibitor (PKRI) were assessed for their impact on both tyrosine phosphorylation (P-Tyr), SphK1 phosphorylation (P-SphK1), and PKR phosphorylation (P-PKR) fluorescence. (**a**) The immunoblotting analysis demonstrates decreased P-SphK1 and corresponding P-Tyr levels in capacitated samples treated with PKRI. (**b** and **c**) Immunocytochemistry analysis shows an increase in P-PKR activation upon induction of capacitation as measured by mean fluorescence (a.u.). For immunocytochemistry analysis, 200 cells were analysed per permeabilized sperm sample (scale bar = 5 μM). The results represent sperm samples from different healthy donors (n = 4, ANOVA and Tukey test; *P≤0.05; **P≤0.01; ***P≤ 0.001).

Since SphK1 is a substrate for PKR, a kinase known to respond to cytokines, ROS, and cellular stress signals that promote apoptosis (Qiao *et al.*, 2021), we incubated spermatozoa with the capacitating agents, FCSu, Sph, or Cer, and the PKR inhibitor. We found a significant reduction of P-SphK1 and P-Tyr levels, indicating the participation of PKR in lipid signalling during sperm capacitation by activating SphK1 (Fig. 9a). We further validated, using immunocytochemistry, the activation of PKR in response to 10 μM Sph and 40 μM Cer. Upon treating samples with FCSu, Sph, and Cer, we observed an increase in P-PKR levels compared to non-treated controls (Fig. 9b). Hence, PKR is an activator of SphK1 during human sperm capacitation.

## Discussion

The present study is the first to characterize the novel signalling cascades of key sphingolipid metabolites (e.g. Sph, Cer, S1P, C1P) during human sperm capacitation. Sphingomyelin and ceramide are the most abundant sphingolipids found in both the seminal plasma and sperm plasma membrane (Furse *et al.*, 2022). We demonstrated that the extracellular addition of Sphingosine or Ceramide promotes tyrosine phosphorylation and makes the spermatozoon responsive to progesterone to undergo the acrosome reaction, which is associated with human sperm capacitation.

Sphingolipids are vital constituents of eukaryotic cells, serving as major structural components and mediators in various biological processes, including cell proliferation, survival or apoptosis, motility, angiogenesis, and immune responses (Ancellin *et al.*, 2002; Spiegel and Milstien, 2007; Gault *et al.*, 2010). In human spermatozoa, sphingolipids are highly abundant and embody bioactive signalling properties that facilitate the acquisition of fertilizing capabilities (Cross, 2004; Suhaiman *et al.*, 2010; Vaquer *et al.*, 2020). However, no previous studies have demonstrated and characterized the role of sphingosine and ceramide during human sperm capacitation.

The ‘sphingolipid rheostat’ demonstrates the capacity of S1P/C1P and Sph/Cer to differentially regulate cell growth and survival/apoptotic pathways by modulating opposing signalling pathways. This is based on the findings that the elevation of Cer induces cell growth arrest and apoptosis, whereas S1P production is required for cell proliferation and suppresses ceramide-mediated apoptotic signalling (Newton *et al.*, 2015). Through pharmacological manipulation of the sphingolipid rheostat, we demonstrated that the inhibition of SphK1 and CERK, and their respective production of S1P and C1P, impair sperm capacitation.

SphK1 is predominantly cytosolic and requires phorbol esters and calcium to facilitate its translocation to the inner leaflet of the plasma membrane (Thompson *et al.*, 2005; Chan and Sieburth, 2012). Another player reported to be involved in the translocation of SphK1 is C1P, which has multiple binding sites on SphK1 and facilitates its translocation (Nishino *et al.*, 2019). We confirmed for the first time in human spermatozoa that the inhibition of CERK impaired the activation of SphK1 (Fig. 3a and b). Despite the unknown mechanism by which C1P participates in the translocation of SphK1 (Nishino *et al.*, 2019), this finding provides evidence that crosstalk occurs when C1P production is fundamental to generating S1P.

SphK1 requires phosphorylation at Ser225 to be activated. Several kinases have been reported to phosphorylate SphK1, including PKC (Johnson *et al.*, 2002); however, the kinase involved remains elusive (Pitson *et al.*, 2003). We demonstrated that the PLC pathway participates in sphingolipid-signalling during human sperm capacitation by promoting PKC-dependent phosphorylation of SphK1. In addition, PKR is activated by auto-phosphorylation of Thr446 and Thr451 in the C-terminal activation loop. In somatic cells, PKR activation is triggered in response to cell stressors such as oxidative stress, cytokines and mycotoxins, which may promote cell death or apoptosis (Qiao *et al.*, 2021). For the first time in spermatozoa, we localized and demonstrated activation of PKR in response to sphingolipids.

One of the first events during sperm capacitation is the production of $O_{2}^{\mathrm{•-}}$ by an as yet undiscovered sperm oxidase at the plasma membrane (O’Flaherty, 2015). Human spermatozoa do not contain NADPH oxidases 1, 2, or 4 but possess NOX5, which is located in the sperm flagellum (Musset *et al.*, 2012) and yield $O_{2}^{\mathrm{•-}}$ production for motility but not capacitation (Serrander *et al.*, 2007; de Lamirande and Lamothe, 2009; Musset *et al.*, 2012). The activation of NOX5 requires the increase of intracellular pH that occurs in capacitating spermatozoa; thus, NOX5 cannot be the source of the initial $O_{2}^{\mathrm{•-}}$ production at the beginning of capacitation (Serafini and O’Flaherty, 2022). However, NOX5 has been associated with oxidative stress, a known culprit of male infertility, since over-expression of NOX5 was observed in asthenozoospermia (Vatannejad *et al.*, 2019).

The fact that the addition of SOD to the capacitating medium impaired P-Tyr induced by Sph or Cer (Fig. 8a) and spermatozoa treated with Sph or Cer produce $O_{2}^{\mathrm{•-}}$ in a similar fashion as FCSu (Fig. 8b) indicates that these sphingolipids promote the activation of the sperm oxidase to generate $O_{2}^{\mathrm{•-}}$ and initiate the onset of sperm capacitation. Then, it is plausible that PKR is activated in response to the spike of $O_{2}^{\mathrm{•-}}$ triggered by sphingolipids. Moreover, one of the pre-established targets of PKR is SphK1 (Qiao *et al.*, 2021). We also demonstrated that P-PKR is a key enzyme in the activation of SphK1, the activation of downstream S1PR1-G_i_ pathways, and capacitation. This groundbreaking interaction sheds light on the novel role of PKR during capacitation and could pave the way for future research in this area.

Following suit with the accepted model that S1P binds to one of its five S1PRs on the outer leaflet (Hla and Brinkmann, 2011), we found that extracellular S1P can promote P-Tyr (Fig. 4a), indicating that S1P binds to the extracellular domain of S1PR1. Since S1P and C1P are produced intracellularly and the interaction of S1P occurs on the extracellular leaflet, S1P requires active transport to the outer leaflet to engage with the S1PR1. Although it has been alluded to in human spermatozoa (Suhaiman *et al.*, 2010), we reported for the first time that ABCC1 is partially responsible for mediating S1P efflux required for capacitation-associated sperm modification. While we could not assess the involvement of SPNS Lysolipid Transporter 2 (SPNS2) due to the lack of a selective inhibitor, we cannot rule out its participation in our proposed pathway mediating S1P efflux (Raggers *et al.*, 2002; Kawahara *et al.*, 2009; Suhaiman *et al.*, 2010).

An important piece of missing information in the S1P signalling in spermatozoa is the identification of the S1P receptor(s) involved in sperm capacitation and acrosome reaction. In the study, both receptors were found in human spermatozoa. S1PR1 was widely distributed and translocated to the post-acrosomal region only during capacitation, and S1PR3 was localized in the post-acrosomal region of the sperm head in non-treated and capacitated samples. According to Cross (2004) and Suhaiman *et al.* (2010), the distribution patterns of specific lipid microdomains undergo reorganization while others remain unchanged and SphK1 redistributes to the acrosomal region before acrosome reaction. This may indicate that the varying distribution patterns of the S1PRs in human spermatozoa pertain to them being differentially regulated by heterogeneous lipid microdomains. To further solidify this claim, we established that S1PR1, not S1PR3, is unequivocally responsible for mediating S1P signal transduction during human sperm capacitation. Additionally, previous work has shown that extracellular S1P could induce acrosome reaction in capacitated spermatozoa; however, the specific S1PR involved was not elucidated (Suhaiman *et al.*, 2010). Here we demonstrated that S1PR3 plays an important role in mediating the acrosome reaction following capacitation (Fig. 5b). Furthermore, these are the first findings to establish that S1PR1 and S1PR3 have different regulatory mechanisms during capacitation and acrosome reaction but are crucial for successful fertilization.

The PI3K-AKT pathway is an important component of sperm capacitation (Luconi *et al.*, 2004; Nauc *et al.*, 2004), but its regulation remains to be fully elucidated. Our findings demonstrate that the activation of PI3K relies on the activation of S1P signalling (Fig. 5c and d). It is known that NOS is responsible for producing NO^•^ as a downstream effector of the PI3K-AKT pathway (Herrero *et al.*, 2003). It was previously demonstrated that the exogenous addition of spermine NONOate, a donor of NO^•^, promoted capacitation-associated modifications in human spermatozoa in the presence of wortmannin (inhibitor of PI3K) and the AKT inhibitor (O’Flaherty *et al.*, 2006b), suggesting that NO^•^ production is downstream PI3K/AKT pathway during capacitation. We found that the inhibition of NOS (Fig. 7a) and the addition of SOD (Fig. 8a) prevented the increase in P-Tyr induced by sphingolipids and that Sph or Cer can promote NO^•^ (Fig. 7b) and $O_{2}^{\mathrm{•-}}$ (Fig. 8b) production in human spermatozoa. These findings indicate that Sph and Cer regulate the activation of the sperm oxidase (by an unknown mechanism) and regulate the PI3K/AKT pathway to ensure NO^•^ production for sperm capacitation, highlighting a novel mechanism that sphingolipids promote capacitation-associated modifications in human spermatozoa.

**Figure 10.**
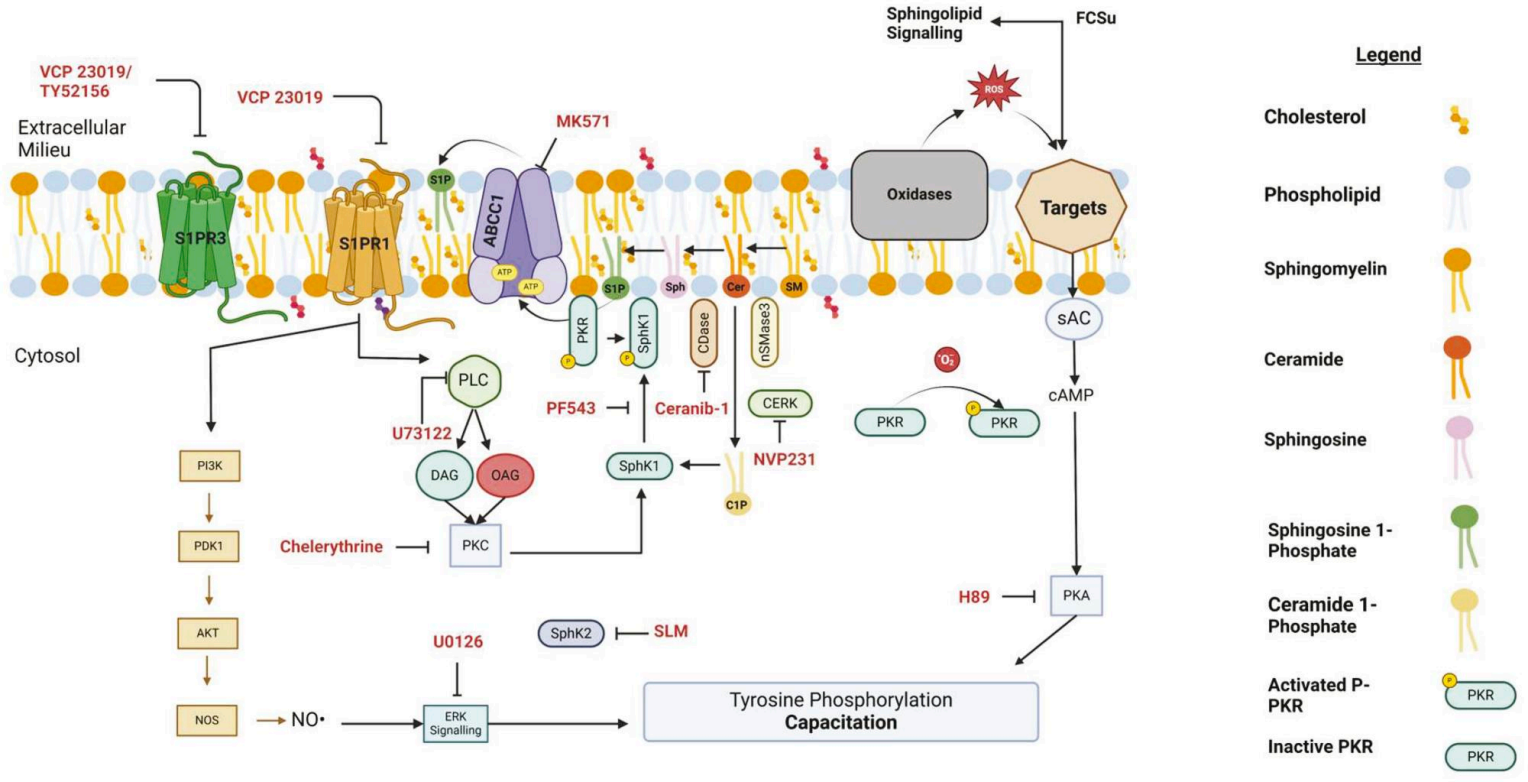
**Schematic of the sphingolipid signalling cascade during capacitation.** Following the production of the bioactive sphingolipid, ceramide (Cer), through the action of a neutral sphingomyelinase (nSMase), Cer will engage with both the ceramidase (CDase) and ceramide kinase (CERK), yielding sphingosine (Sph), and ceramide 1-phosphate (C1P), respectively. Sphingosine (sph) and Cer will lead to a spike in superoxide ($O_{2}^{\mathrm{•-}}$) production by the sperm oxidase and protein kinase R (PKR) will auto-phosphorylate (P-PKR). C1P, PKC, and P-PKR will facilitate the translocation of SphK1 to the inner leaflet of the plasma membrane and activation by phosphorylation (Ser^225^). P-SphK1 will convert Sph to sphingosine 1-phosphate (S1P), and the latter will interact with the ABCC1 transporter and efflux to the outer leaflet. S1P will interact with the S1PR1 and initiate the G_i_ signalling cascade. Signal transduction activates the PI3K-AKT and phospholipase C (PLC)-PKC pathways that lead to the subsequent activation of nitric oxide synthase (NOS) and nitric oxide (NO^•^) production and SphK1, respectively. NO^•^ interacts with Ras to facilitate the extracellular signal-regulated kinase (ERK) signalling cascade leading to an increase in tyrosine phosphorylation (P-Tyr) of prominent sperm proteins required for capacitation. The figure was created with BioRender.com.

We have proposed a model that explains the molecular mechanisms involved in the sphingolipid-dependent signalling during human sperm capacitation (Fig. 10). The supplementation of human spermatozoa with Sph or Cer promotes the incorporation of these sphingolipids into the plasma membrane. These sphingolipids are then converted into S1P and C1P by P-SphK1 and CERK, respectively. Ceramide is converted into Sph by the action of Ceramidase. Once formed, S1P is translocated to the outer leaf of the plasma membrane by the participation of the ABCC1 transporter. S1P then binds to the Gi-coupled S1PR1, inducing the activation of the PI3K-AKT to promote NO^•^ production and activating the ERK pathway by engaging with Ras during human sperm capacitation (O’Flaherty *et al.*, 2006b). Our findings show that the S1P signalling requires the participation of PLC and PKC pathways to translocate and activate the SphK1 by phosphorylation. Additionally, the activation of SphK1 depends on the activation of PKR by ROS ($O_{2}^{\mathrm{•-}}$ and or H_2_O_2_) generated at the beginning of capacitation (de Lamirande *et al.*, 2009; O’Flaherty, 2015; Puga Molina *et al.*, 2018). Our findings indicate that the S1P-S1PR1-Gi signalling modulates capacitation independently of the cAMP/PKA pathway, which suggests that multiple signalling pathways are required to support human sperm capacitation (Serafini and O’Flaherty, 2022).

Male infertility is a major health issue, and in about 34% of cases, the cause remains unknown. It is important to note that the conventional method of semen analysis is limited in its ability to measure the functional capacity of sperm in a sample, which restricts its ability to assess its potential to fertilize an oocyte. Further elucidating the fundamental components that are required for the spermatozoa to capacitate is pivotal for understanding the pathologic mechanisms driving male infertility. The sperm of healthy males with impaired fertility may have altered sphingolipid profiles (Schisterman *et al.*, 2014; Craig *et al.*, 2019). We found that supplementing the capacitation medium (containing FCSu) with Sph or Cer promoted capacitation-associated modifications in spermatozoa isolated from the 40% layer of the Percoll density gradient, which display reduced motility and an inability to capacitate (Aitken and West, 1990). Hence, this proof-of-concept experiment supports the potential use of sphingolipid supplementation to promote capacitation and improve *in vitro* fertilization or artificial insemination to treat male infertility.

In conclusion, this study unravelled the novel role of sphingolipids, particularly the bioactive lipid S1P, during human sperm capacitation, the essential last maturational step for the capacity of the spermatozoon to undergo the acrosome reaction and ultimately fertilize the oocyte. The data presented stress the importance of sphingolipid metabolism and impairments that can disturb the lipid balance and may compromise a male’s fertility status. Our findings lay the foundation for further exploration of the role of bioactive sphingolipid metabolites during human sperm capacitation. Ultimately, the data presented may reveal new insights into the signalling cascades that could impact male infertility, bridging the gap between basic research and translational medicine.

## Supplementary Material

deae268_Supplementary_Figure_S1

deae268_Supplementary_Figure_S2

deae268_Supplementary_Figure_S3

deae268_Supplementary_Figure_S4

deae268_Supplementary_Figure_S5

## Data Availability

There are no database-deposited data in this article. All relevant data are available from the authors.
