# Supplementary material for: Sphingolipids modulate redox signalling during human sperm capacitation
Source: Hum Reprod. 2024 Dec 10;40(2):210–25. doi: 10.1093/humrep/deae268 (PMC11788196; doi:10.1093/humrep/deae268)
Supplement: deae268_Supplementary_Figure_S1 [file deae268_supplementary_figure_s1.pdf]

OXFORD
human reproduction

Human Reproduction, 2024, 1–1
https://doi.org/10.1093/humrep/deae268
Supplementary Data

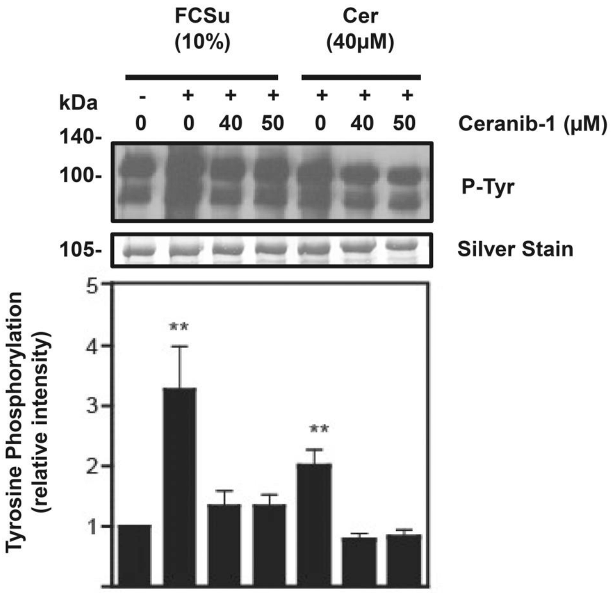

Supplementary Figure S1. Conversion of Cer to Sph is fundamental for sequential sphingolipid signalling. Foetal cord serum ultrafiltrate (FCSu)- and ceramide (Cer)-capacitated spermatozoa incubated with or without ceramidase inhibitor (Ceranib-1) were assessed for their impact on tyrosine phosphorylation (P-Tyr) fluorescence. Immunoblotting demonstrates the decrease in P-Tyr levels in capacitated samples treated with 40 and 50 μM of Ceranib-1. The results represent sperm samples from different healthy donors ($n = 4$, ANOVA and Tukey test; $^{**}P \leq 0.01$).
