# Supplementary material for: Sphingolipids modulate redox signalling during human sperm capacitation
Source: Hum Reprod. 2024 Dec 10;40(2):210–25. doi: 10.1093/humrep/deae268 (PMC11788196; doi:10.1093/humrep/deae268)
Supplement: deae268_Supplementary_Figure_S2 [file deae268_supplementary_figure_s2.pdf]

OXFORD
human reproduction
Human Reproduction, 2024, 1–1
https://doi.org/10.1093/humrep/deae268
Supplementary Data

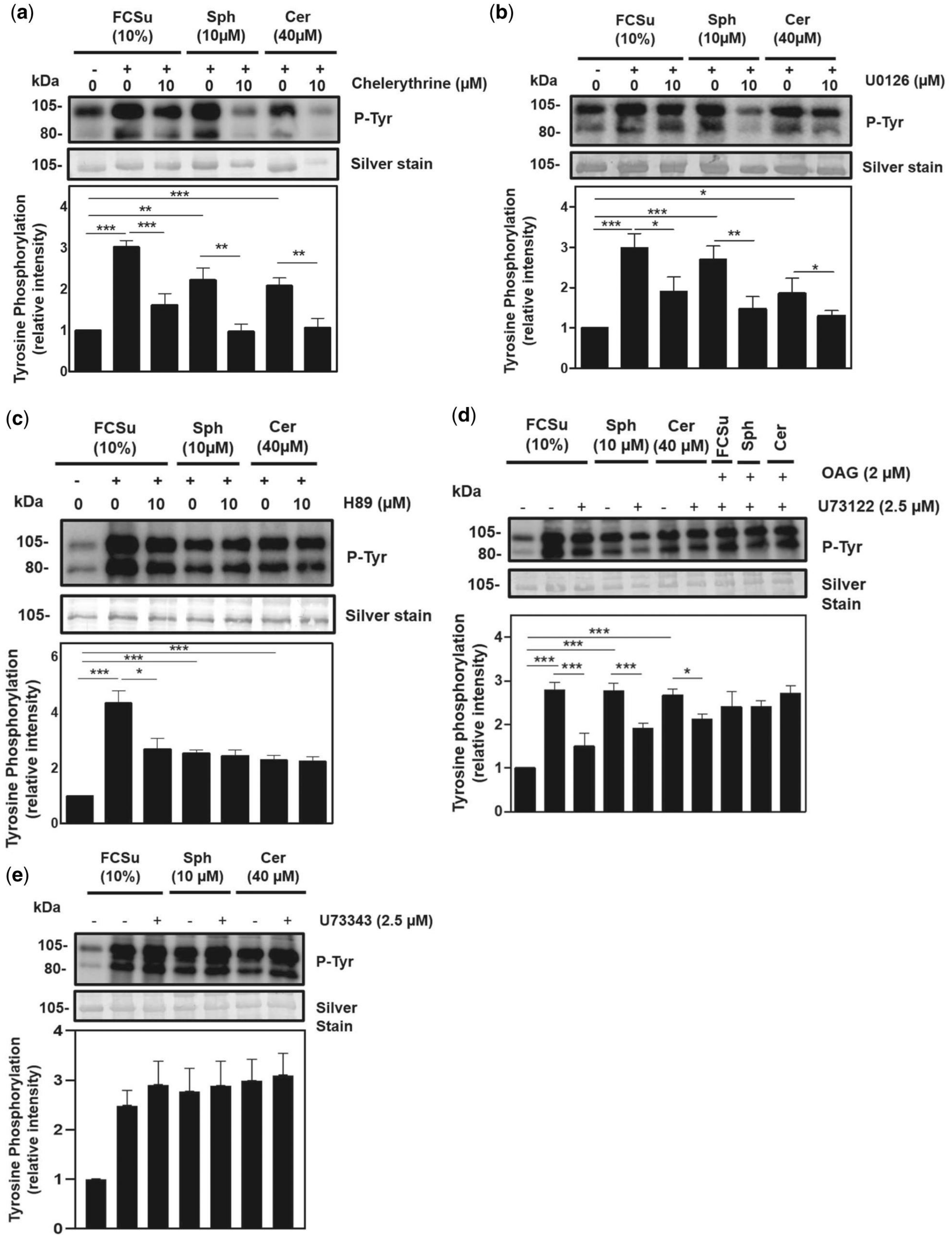

Supplementary Figure S2. Protein kinases are associated with sphingolipid signalling during capacitation. Foetal cord serum ultrafiltrate (FCSu)-, sphingosine (Sph)-, and ceramide (Cer)-capacitated spermatozoa incubated with or without (**a**) protein kinase C (PKC) inhibitor (Chelerythrine), (**b**) extracellular signal-regulated kinase (ERK) inhibitor (U0126), (**c**) protein kinase A (PKA) inhibitor (H89), (**d**) phospholipase C (PLC) inhibitor (U73122), and (**e**) (U73343) an inactive cell-permeable analog of U73122 were assessed for their impact on tyrosine phosphorylation (P-Tyr) fluorescence. (a, b, d) Immunoblotting demonstrates decreased P-Tyr levels in capacitated spermatozoa treated with 10 μM Chelerythrine, 10 μM U0126, and 2.5 μM U73122, respectively. (d) The addition of 2 μM of OAG prevented the reduction of P-Tyr levels by inhibiting PLC with U73122. (e) The addition of 2.5 μM of U73343 (inactive analog of U73122 used as negative control) did not impair the P-Tyr level. The immunoblotting analysis demonstrates no change in P-Tyr immunoblot in capacitated samples treated with 10 μM of H89 (c). The results represent sperm samples from different healthy donors (n = 4, ANOVA and Tukey test; $*P \leq 0.05$; $**P \leq 0.01$; $***P \leq 0.001$).
