# Supplementary material for: Sphingolipids modulate redox signalling during human sperm capacitation
Source: Hum Reprod. 2024 Dec 10;40(2):210–25. doi: 10.1093/humrep/deae268 (PMC11788196; doi:10.1093/humrep/deae268)
Supplement: deae268_Supplementary_Figure_S3 [file deae268_supplementary_figure_s3.pdf]

OXFORD
human reproduction
Human Reproduction, 2024, 1–1
https://doi.org/10.1093/humrep/deae268
Supplementary Data

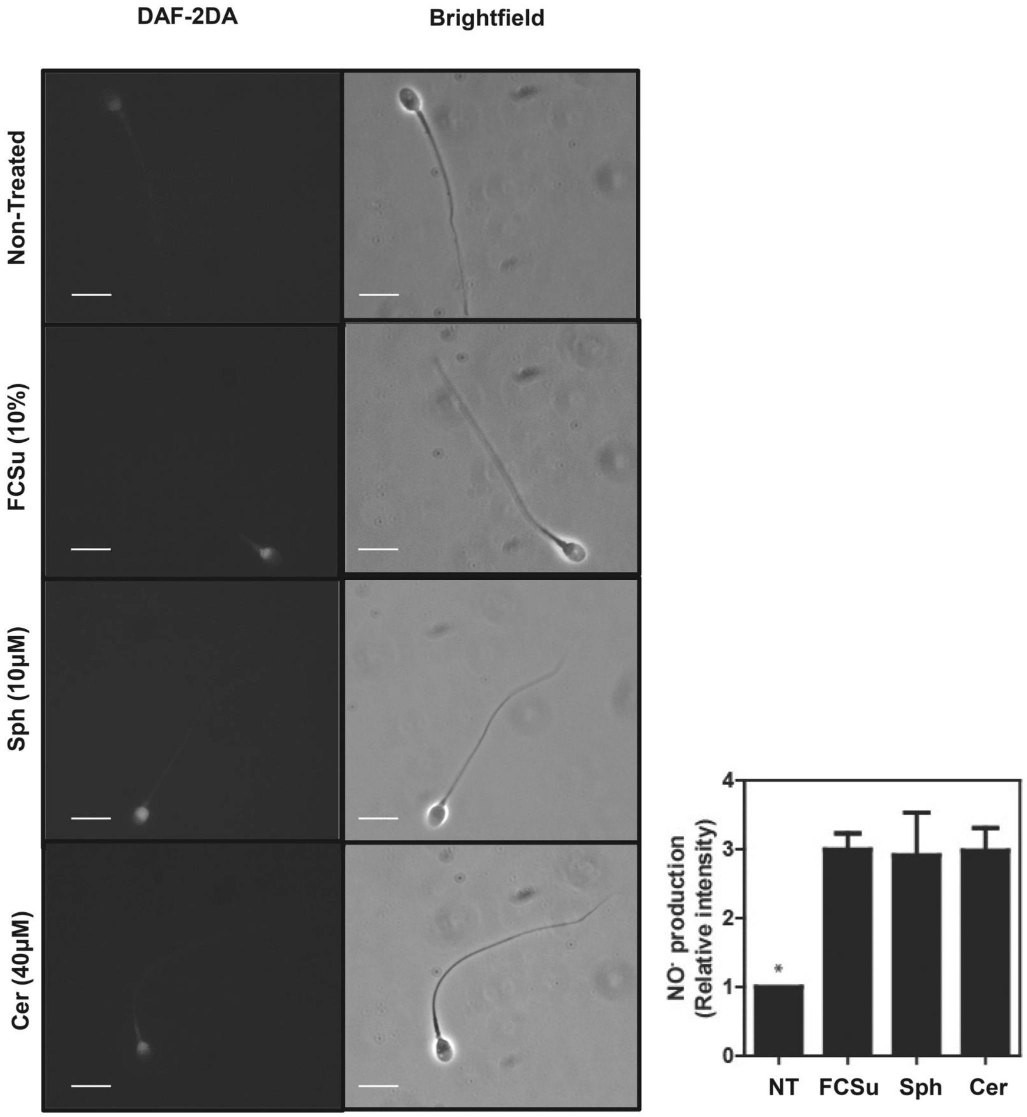

Supplementary Figure S3. Localization of NO· production during sphingolipid-induced human sperm capacitation. Highly motile spermatozoa were pre-treated with DAF-2DA. After they were incubated with or without foetal cord serum ultrafiltrate (FCSu)-, sphingosine (Sph)-, and ceramide (Cer). The increase in nitric oxide (NO·) levels during capacitation and the location of the production in the post-acrosomal region of the sperm head were determined using fluorescence microscopy (scale bar = 5 µM). Localization of NO· was assessed through the obtainment of 200 cells per sample. The results represent sperm samples from different healthy donors (n = 4, ANOVA and Tukey test; $*P \leq 0.05$).
