# Supplementary material for: Sphingolipids modulate redox signalling during human sperm capacitation
Source: Hum Reprod. 2024 Dec 10;40(2):210–25. doi: 10.1093/humrep/deae268 (PMC11788196; doi:10.1093/humrep/deae268)
Supplement: deae268_Supplementary_Figure_S4 [file deae268_supplementary_figure_s4.pdf]

OXFORD

human reproduction

Human Reproduction, 2024, 1–1

https://doi.org/10.1093/humrep/deae268

Supplementary Data

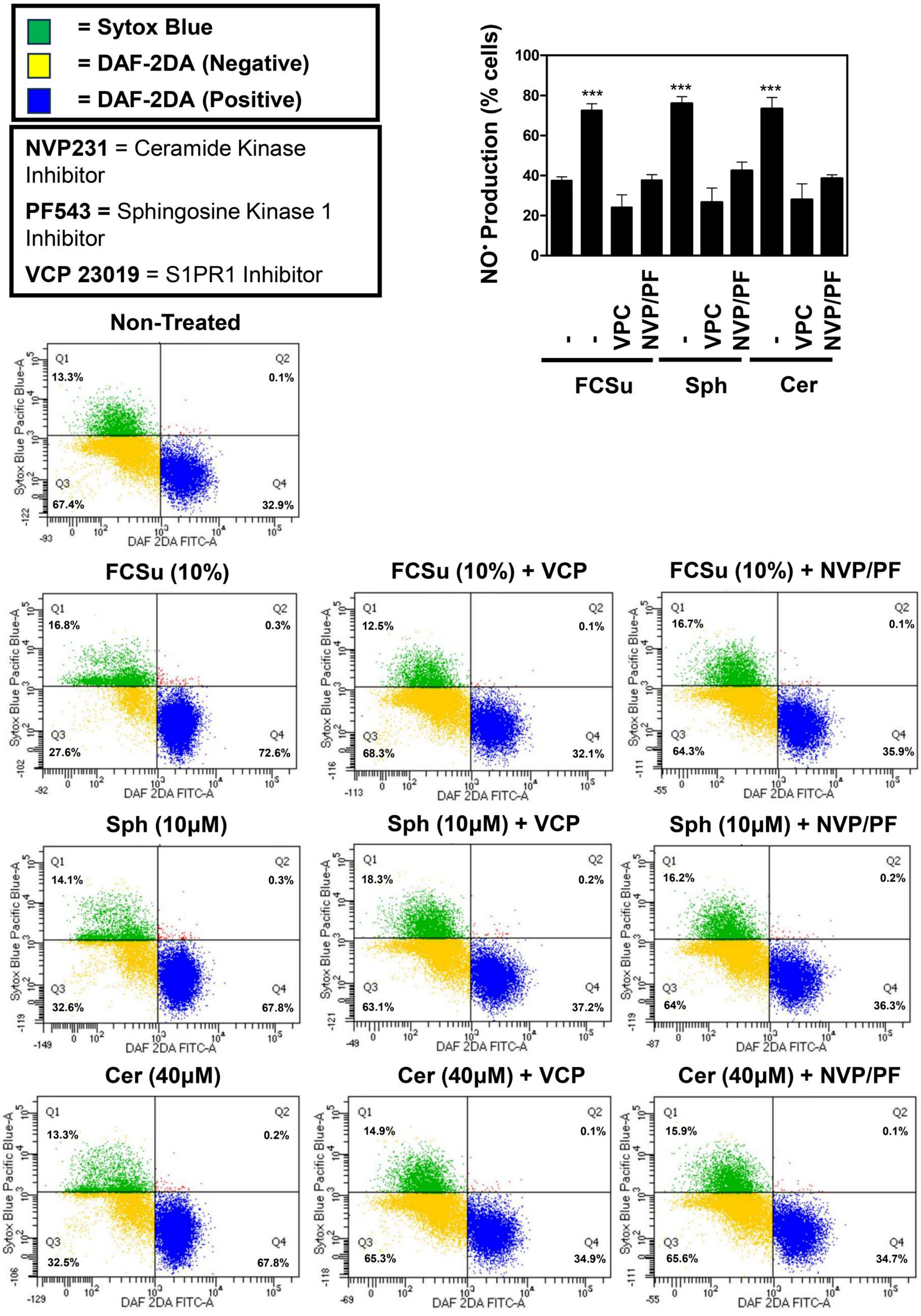

Supplementary Figure S4. NO· production by live spermatozoa during capacitation. Representative dot plots (from experiment shown in Fig. 5) showing the production of nitric oxide (NO·) by spermatozoa treated with foetal cord serum ultrafiltrate (FCSu)-, sphingosine (Sph)-, and ceramide (Cer)-capacitated spermatozoa incubated with or without S1PR1 inhibitor (VCP23019) or CERK inhibitor (NVP231) and SphK1 inhibitor (PF543). Live spermatozoa (Sytox Blue negative) that are either DAF2-DA$^+$ (blue, quadrant Q4) or DAF2-DA$^-$ (yellow, quadrant Q3) for NO· production. Quadrants Q1 and Q2 represent spermatozoa Sytox blue$^+$ (dead cells) and DAF2-DA$^-$ (green) or DAF2-DA$^+$ (red), respectively. The percentages of spermatozoa in each quadrant were added. Note that the population of dead spermatozoa (shown in red in Q2) are lower than ≤0.3% in all cases. Nitric oxide production is represented in the bar graph as a percentage of live spermatozoa producing NO· (n = 4, ANOVA and Tukey test; ***P ≤ 0.001).
