# Supplementary material for: Sphingolipids modulate redox signalling during human sperm capacitation
Source: Hum Reprod. 2024 Dec 10;40(2):210–25. doi: 10.1093/humrep/deae268 (PMC11788196; doi:10.1093/humrep/deae268)
Supplement: deae268_Supplementary_Figure_S5 [file deae268_supplementary_figure_s5.pdf]

Human Reproduction, 2024, 1–1
https://doi.org/10.1093/humrep/deae268
Supplementary Data

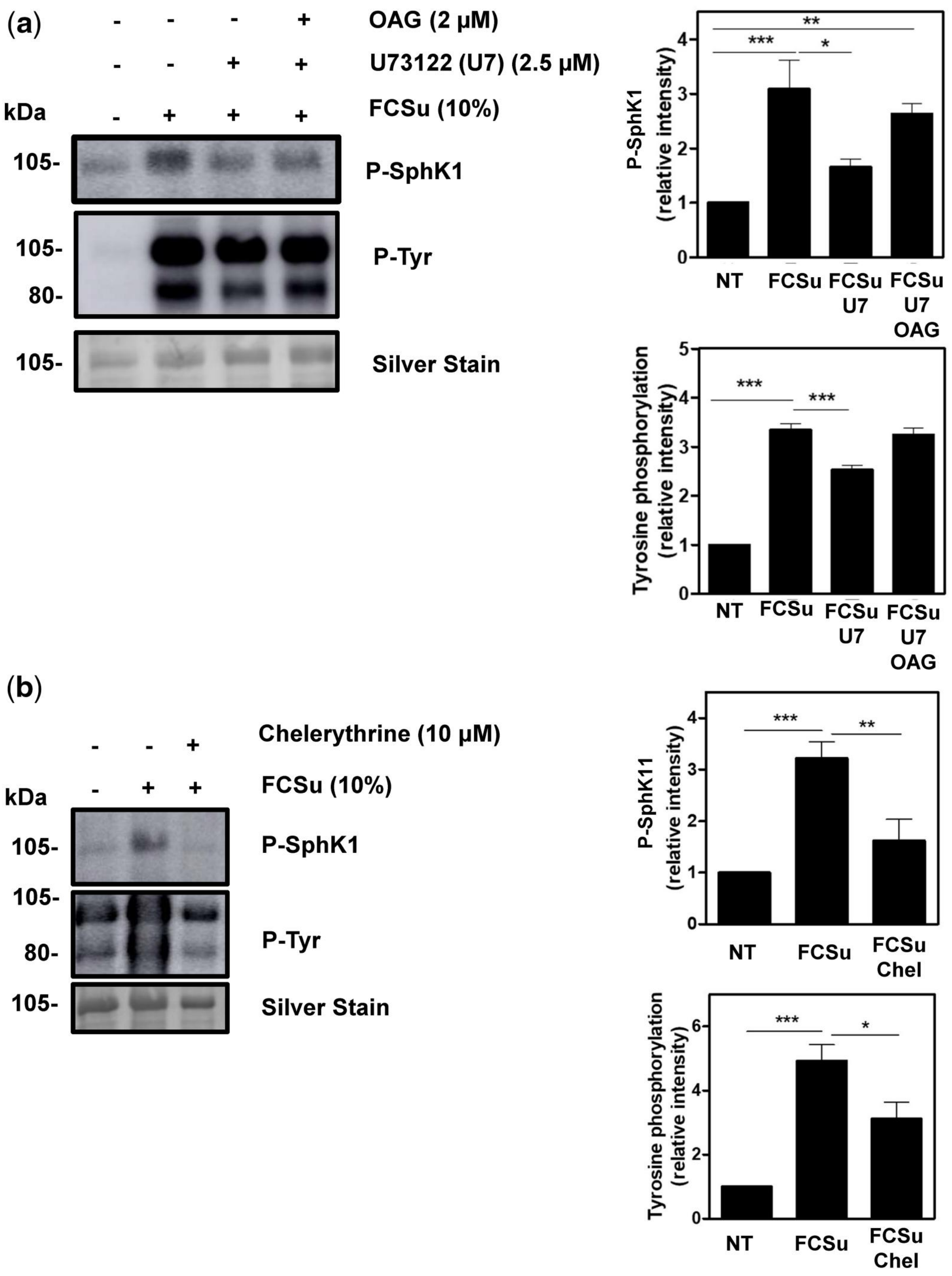

Supplementary Figure S5. Activation of PLC leads to PKC and subsequent activation of SphK1 activation. Foetal cord serum ultrafiltrate (FCSu) capacitated spermatozoa incubated with or without (**a**) phospholipase C (PLC) inhibitor (U73122) and (**b**) protein kinase C (PKC) inhibitor (Chelerythrine) were assessed for their impact on both tyrosine phosphorylation (P-Tyr) and SphK1 phosphorylation (P-SphK1) fluorescence. (a) Immunoblotting demonstrates the decrease in both P-SphK1 and corresponding P-Tyr levels in capacitated samples treated with U73122, followed by recovery with the addition of 1-Oleoyl-2-acetyl-sn-glycerol (OAG). (b) Immunoblotting demonstrates decreased P-SphK1 and corresponding P-Tyr levels in capacitated spermatozoa treated with Chelerythrine (10 μM). The results represent sperm samples from different healthy donors (n = 4, ANOVA and Tukey test; $*P \leq 0.05$; $**P \leq 0.01$; $***P \leq 0.001$).
